# Traditional music, recovery-oriented psychological functioning, and psychological resilience among ethnic minority communities in Southwest China: a sequential explanatory mixed-methods study

**DOI:** 10.3389/fpsyg.2026.1932535

**Published:** 2026-09-03

**Authors:** Haini Wu

**Affiliations:** School of Music, Leshan Normal University, Leshan, Sichuan, China

**Keywords:** ethnic minorities, perceived social support, psychological resilience, Southwest China, traditional music, trauma recovery

## Abstract

**Introduction:**

Traditional music is an integral component of cultural identity, collective participation, emotional expression, and social relationships among ethnic minority communities in Southwest China. However, the pathways through which Traditional Music Engagement (TME) may relate to recovery-oriented psychological functioning (ROPF), Perceived Social Support (PSS), and Psychological Resilience (PR) remain insufficiently understood. This study examined the associations among TME, Cultural Identity (CI), Community Participation (CP), Emotional Expression through Music (EEM), ROPF, PSS, and PR among members of the Dai, Yi, Miao, and Dong communities.

**Methods:**

A sequential explanatory mixed-methods design was employed. Quantitative data were collected from 503 participants and analyzed using confirmatory factor analysis (CFA), structural equation modeling (SEM), and bias-corrected bootstrapping with 5,000 resamples. Subsequently, 32 adult participants were interviewed to contextualize and elaborate on the quantitative findings. Qualitative data were analyzed thematically, and the quantitative and qualitative findings were integrated during interpretation.

**Results:**

The measurement model demonstrated satisfactory fit (CFI = 0.961, TLI = 0.956, RMSEA = 0.051), and the structural model showed good fit (CFI = 0.955, TLI = 0.951, RMSEA = 0.054). TME, CI, CP, and EEM were positively associated with ROPF and PSS. ROPF (*β* = 0.421, *p* < 0.001) and PSS (*β* = 0.288, *p* < 0.001) were positively associated with PR, with the model explaining 73.4% of the variance in PR. The qualitative analysis generated four interrelated themes: emotional expression and regulation; cultural continuity and meaning; collective support and community cohesion; and adaptive resilience and future orientation.

**Discussion:**

Integrated findings suggest that traditional music may function as a culturally embedded emotional, cultural, and relational resource associated with recovery-oriented psychological functioning, perceived social support, and psychological resilience among ethnic minority communities in Southwest China. The qualitative findings further indicate that music engagement may support emotional regulation, cultural continuity, collective connectedness, and adaptive orientations toward the future. Nevertheless, the cross-sectional and self-reported nature of the quantitative data limits causal interpretation. Future longitudinal and multimethod research is warranted to examine the temporal and causal pathways underlying these associations.

## 1 Introduction

Psychological adaptation is shaped by how individuals and communities respond to adversity, maintain social relationships, construct meaning, and mobilize culturally available resources. In culturally diverse communities, these processes may be embedded in everyday practices rather than confined to formal psychological or clinical interventions. In turn, there has been a growing interest in culturally relevant resources that could be used to complement formal mental health services to facilitate coping, adaptation, social engagement, and psychological resilience. Traditional Music is one such resource. Whilst music can be used to express emotion, entertain or simply enrich self-expression, it may also serve as a medium through which collective memory, cultural values, interpersonal relationships, and responses to hardship are expressed and maintained (Gwerevende & Mthombeni, 2023; Ma, 2022). Communities can use songs, rituals, ceremonies, call to memory, festival, and everyday musical interaction to share experiences that are difficult to communicate directly while maintaining cultural continuity and social belonging (Sumiaty et al., 2023).

The term ethnic minority communities is used to describe the Dai, Yi, Miao and Dong based on the terminology typically used in Chinese scholarship and the historical and sociopolitical context of China. International research on Indigenous resilience can provide valuable concepts to consider for cultural continuity, collective identity, community strengths and culturally responsive well-being. These frameworks are not however regarded as directly applicable to the experience of ethnic minorities in Southwest China, as the experience of colonisation, dispossession, sovereignty, or settler imperialism cannot be simply mapped across time. Their relevance is thus seriously analyzed and interpreted in the context of the cultural, historical and contemporary social context of the Dai, Yi, Miao and Dong communities that is studied here.

Ethnic and cultural diversity are prominent in southwest China, and music plays a significant role in the cultural life of many ethnic minority groups, such as the Dai, Yi, Miao and Dong (D’Amico, 2024; Guo, 2024). Traditional music practices are still significant in many communities, and continue to play a role despite urbanisation, migration, technological change, economic change and cultural exchange. Folk songs, ritual songs, ceremonial songs, festival songs, instrumental traditions, intergenerational learning, and ritual practices and songs can offer opportunities for collective participation, social interaction, knowledge sharing, and cultural expression. In fact, traditional music is not only an integral part of the performance but also of the family relationship, community life, collective memory and cultural continuity (Liu & Song, 2025). However, there are few empirical studies that provide an explanation of the relationship between engagement in these practices and recovery-oriented psychological functioning, perceived social support, and psychological resilience in ethnic minority communities in Southwest China (Smye et al., 2023; Ho 2025 and Yang et al., 2025).

Previous studies have found positive correlations between music engagement, emotional regulation, psychological well-being, coping and resilience in various populations (Dudgeon and Bray, 2024). Research on music therapy and music interventions suggests that music-related tasks can help individuals feel their emotions, cope with psychological stress, enhance interpersonal relationships, and foster adaptive coping mechanisms in challenging situations (Alexandre et al., 2025; Feng & Wang, 2025; Guo, 2025; Han et al., 2024; Wang et al., 2024). The significance of cultural identity, community engagement, and social support as sources of psychological adaptation and societal health is also emphasized in the research on culturally embedded music (Hanif & Sri Maruti, 2024; Netshivhambe, 2025; Zhou et al., 2025). Rather, many of the previous studies have investigated those factors individually, or studied clinical populations, university students, bereaved individuals, or conflict-affected groups, but not the musical practices of ethnic minority communities that are embedded in the everyday cultural activities of their life (Alexandre et al., 2025; Guo, 2025; Han et al., 2024; Lai et al., 2025).

Traditional music participation cannot be confused with clinical music therapy. Typical clinical music therapy includes planned therapeutic goals, the intervention procedure is structured, and clinical and/or psychosocial outcomes are defined, with professional facilitation. Contrary to that, the musical practices discussed in this study are culture-laden and natural activities that take place within community events and festivals, rituals, intergenerational transmission, memory and social engagement. The psychological significance of music experience may therefore emerge through other aspects of music such as cultural significance, identity affirmation, joint participation, intergenerational continuity and the relationship between people. The current research, therefore, examines traditional music as an ingrained cultural psychosocial resource, rather than a replacement for professionally administered clinical treatment.

There is also no such thing as traditional music being a uniform type of cultural engagement. Folk songs can help to communicate emotion, autobiographical significance, and narrative coping; ceremonial chants can ensure ritual meaning and the continuation of the past; festival music can foster social bonding and a sense of community; and ensemble music can promote collaboration, synchronisation and shared success. The transmission of music across generations can help to perpetuate cultural learning, identity continuity and responsibility for the next generation. This current study will focus on the common factors instead of giving individual estimates of effect for each musical subtype. The results therefore give a general picture of what folk, ceremonial, festival, instrumental, or community-based practices of music engagement are like, but do not indicate whether they work in the same way.

Conceptually, the work has tended to separate out measurable psychological relationships from the lived experience of participants in previous studies, methodologically. Interventions designs, longitudinal designs, mediation analysis and structural equation modelling (SEM) have been used to investigate relationships between cultural involvement, mental health and resilience (Feng & Wang, 2025; Guo, 2025; Wang, et al., 2024; Zhou, et al., 2025). Phenomenology, ethnography, grounded theory and other qualitative methods have been used in qualitative studies of cultural meanings and personal experiences (Hanif & Sri Maruti, 2024; Lai et al., 2025; Li et al., 2025; Myers-Coffman, 2024; Netshivhambe, 2025). Both methods offer useful information, but they are not sufficient to describe the possible interaction of emotional, cultural and relational processes in traditional musical practices. Mixed methods research can thus offer a more holistic perspective in that it allows for statistical connections to be explored and then “put in context” with the participants’ own accounts.

In this study the researchers investigate the relationships between Traditional Music Engagement, Cultural Identity, Community Participation, Emotional Expression through Music, and recovery-oriented psychological functioning and Perceived Social Support and explore the relationship between these constructs and Psychological Resilience. This paper also examines whether recovery-oriented psychological functioning and perceived social support statistically account for indirect associations between the traditional music-related variables and Psychological Resilience. Because all quantitative measures were collected cross-sectionally, these indirect associations are interpreted as covariance patterns consistent with the proposed model rather than evidence of temporal mediation. Because trauma exposure was not used as an inclusion criterion, the recovery-related construct is defined as recovery-oriented psychological functioning, not as evidence for clinical trauma recovery.

This research is designed to achieve five specific goals: explore the connections between traditional music factors and recovery-oriented psychological functioning; explore connections between these factors and perceived social support; explore connections between recovery-oriented psychological functioning and perceived social support and psychological resilience; test the proposed indirect relationships between these constructs through a structural model; and interpret the quantitative results using data from qualitative research. Existing resilience frameworks frequently conceptualize adaptive functioning primarily at the individual psychological level, whereas culturally embedded practices may organize emotional expression, cultural continuity, social participation, and support simultaneously. The present study therefore examines whether traditional music can be understood as a culturally embedded context in which these resources are interconnected. By combining a latent variable modeling approach with narratives from culturally sensitive participants, the study seeks to provide culturally sensitive interpretations of how traditional music can be related to emotional coping, cultural continuity, community involvement, and psychological resilience among Dai, Yi, Miao, and Dong communities in Southwest China.

## 2 Literature review

### 2.1 Music, psychological well-being, and resilience

A recent body of literature suggests that music-based interventions and musical engagement can be beneficial in psychological well-being, emotional regulation, coping and resilience in various populations. Despite the differing theoretical perspectives, intervention designs, participant characteristics, and analyses in these studies, a common theme is that musical activity can serve as a psychosocial resource that people use to express emotion, manage distress, enhance relationships, and respond adaptively to adversity. The significance of music and its possible benefits, however, are not consistent across different populations, different cultural contexts, different musical forms, different social settings and different circumstances (whether music is presented as a therapeutic structure or as an ordinary cultural activity). There is fairly good evidence from intervention studies on the potential benefits of planned programmes of music. An eight-week music therapy intervention was found to enhance emotional resilience and was related to increase in wellbeing and employability, according to Feng and Wang (2025). According to their mediation analysis, well-being partially mediated the relationships between emotional resilience and employability, and age, education moderated parts of the proposed relationships. Guo (2025) also reported that music therapy positively impacts the psychological health, memory, and attention of university students, especially those who are clinically depressed. Han et al. (2024) found that the stress level, anxiety, depression, and post traumatic symptoms were reduced and resilience and psychological capital were improved after the virtual MT intervention. Similar results have been seen with bereaved individuals, conflict, and other challenging life circumstances. Wang et al. (2024) reported that music therapy has a positive effect on psychological recovery for bereaved families, and that music therapy compared with non-music therapy treatments within an optimised approach framework. Vulnerable individuals in conflict affected areas showed a decrease in anxiety, depression and post-traumatic stress symptoms after engaging in songwriting music therapy, which was still present after 6 months. Myers-Coffman (2024) also highlighted that culturally responsive music therapy can potentially address inter-generational, collective, systemic and interpersonal aspects of trauma for bereaved youth. The findings from these studies indicate that music-based interventions could play a role in psychological support and highlight the significance of culture and relational context. These findings provide evidence that music can be relevant to emotional and adaptive functioning under structured clinical conditions, but they cannot be assumed to demonstrate equivalent relationships for naturally occurring traditional music practices embedded in everyday community life; they are treated here as contextual background rather than direct evidence for the effects of traditional music engagement.

### 2.2 Psychological safety and cultural context

The quantitative findings are supported by qualitative and mixed-method studies of music-related practices that could contribute to well-being. Based on music therapists’ experiences in multiple countries around the world, Lai et al. (2025) concluded that psychological safety is a key factor in trauma-informed music therapy. Effective practice was more than just about music and involved the development of well-organised, supportive, participant-centred therapeutic spaces for emotional expression, autonomy, trust and control. This discovery indicates that the therapeutic effects might be related in part to the relational context under which music is experienced, rather than the music itself. To investigate the mental health of Tibetan music teachers and elucidate the dynamic psychological, occupational and sociocultural factors that contribute to positive mental health, Li et al. (2025) adopted a mixed-methods approach. Their qualitative analysis pointed to cultural communication issues, work-related stresses and personal psychological issues. Rather than interventions which mainly focus on evaluating the outcomes of interventions, this research provided evidence that musicians and music teachers may need to be supported psychologically and organisationally at a broader level in culturally diverse contexts. Although methodological variations are present, numerous studies in this literature associate music with psychological recovery and emotional regulation; however, this evidence again derives predominantly from structured clinical or intervention contexts rather than from naturally occurring traditional music practice, and should be read as contextual background accordingly. Important limitations remain. Numerous intervention studies are conducted with clinical populations, university students, bereaved or conflict affected individuals. Very little research is conducted on communities where music is not used as a therapeutic intervention, but rather it is part of the collective life and cultural continuity. However, quantitative studies seldom involve modeling recovery-oriented functioning and perceived social support together, and qualitative studies do not necessarily test hypothesized relationships in a latent-variable model. The constraints underscore the necessity for a combined research strategy involving mixed methods.

### 2.3 Traditional music as a differentiated cultural practice

Music of the past should not be viewed as a uniform experience. Meanings, settings and psychosocial processes may differ across and within musical forms. Folk songs may provide opportunities to express emotion, make sense of personal experiences, communicate difficult experiences, and tell stories that are difficult to put into words. Ceremonial chants may carry ritual significance, religious practice, cultural identity and ancestral or collective memory functions. Festival music may support social cohesion, solidarity, togetherness, community, and public recognition. Group or ensemble work and instrumental playing may involve collective performance, synchrony, and shared responsibility. Intergenerational music transmission may be experienced as a context for cultural learning, identity continuity, and responsibility toward younger generations. Community singing may provide opportunities for emotional co-regulation, belonging, and perceived support. This distinction is important, because similar amounts of musical involvement could carry different psychological meanings depending on the form and context of that involvement. A ceremonial chant is a different kind of practice from a festival ensemble, a family song, or a community singing event: some practices may centre on spiritual or ancestral relationships, others on emotional expression, social collaboration, cultural education, or public belonging. Traditional music may also evoke feelings of sadness, loss, or difficult personal and communal memories; its psychological meaning may therefore be experienced as comforting in some situations and as emotionally activating in others. In the present study, some shared psychosocial dimensions are explored across multiple forms of traditional music, but no attempt is made to estimate separate effects for each musical type. This approach establishes a general framework of culturally embedded music engagement within which emotional expression, cultural identity, community engagement, recovery-oriented functioning, perceived support, and resilience can be examined simultaneously. It remains unknown, however, whether folk songs, ceremonial music, festival music, instrumental music, or intergenerational transmission function in the same way psychologically. This diversity of traditional music should be taken into account when interpreting the results and merits dedicated study in its own right.

Unless one equates traditional music engagement with clinical music therapy, an engagement with traditional music is not a therapy. Generally, clinical music therapy follows a planned therapy goal, a professional facilitation, a structured intervention procedures and a defined clinical or psychosocial outcomes. The traditional music examined in this study are culturally spontaneous activities that are naturally part of their daily life, festivals, ceremonies, family transmission, collective memory, spiritual practice and community participation, in contrast to the traditional music practices of past studies. These potential psychological meanings can thus be musical experience, cultural meaning, shared participation, identity affirmation, intergenerational continuity, and social relationships. In the case of clinical music therapy, evidence is thus relevant as background, and not directly transferrable to traditional cultural practices without taking into account these differences.

### 2.4 Traditional music, cultural identity, and collective well-being

Recent studies indicate traditional and community music potentially has a role in cultural continuity, social integration and psychological well-being. Netshivhambe (2025) reported that those older South Africans who participated in traditional instrumental music reported higher happiness, less stress and depression, and better well-being than those who did not. Hanif and Sri Maruti (2024) found that during the COVID-19 pandemic, the activities of Karawitan helped to build community resilience in the areas of health communications and reinforcement of religious and social values and by providing collective support. These practices were not structured clinical programmes, but were mediated by communicative, participatory and supportive practices embedded in the culture. The research on cultural identity also supports the notion of sociocultural resources as significant factors in psychological adjustment. Zhou et al. (2025) reported that, for Chinese university students, cultural identity was positively related to meaning in life, which was mediated first by perceived social support and then by resilience. The results indicate that cultural affiliation may be associated with psychological functioning both directly and indirectly, through an interpersonal pathway involving perceived social support and adaptive capacities. Culturally meaningful participation may therefore be associated with internal resources, such as purpose and confidence, and external resources, such as belonging and social support. However, what is known does not guarantee that traditional music practices in all communities have the same effects. Music has cultural significance which is determined by history, language, ritual, social organisation and local involvement. Studies based on one tradition should not be taken as a representative of other communities. Each of these ethnic communities, namely Dai, Yi, Miao and Dong, has a unique musical tradition and cultural history, and there may be a large number of cultural specific mechanisms that may not be represented in a broad model of the cross-community. In the present study therefore common psychosocial dimensions are focused on and it is recognized that common measurement does not necessarily mean similar cultural meaning.

### 2.5 Methodological developments and research gaps

Experiments, longitudinal, mixed method, phenomenological, ethnographic, and grounded theory methods have been used in recent research. The quantitative studies have employed intervention-control designs, structural equation modelling, mediation analysis, or longitudinal methodologies for quantifying relationships between psychological variables. Lived experience, cultural meaning and contextual processes have been explored in qualitative studies employing phenomenology, interpretative phenomenological analysis, grounded theory and ethnography. While these advances have contributed to the better knowledge of music and well-being, quantitative and qualitative studies often take place separately, and existing resilience frameworks frequently conceptualize adaptive functioning primarily at the individual psychological level, whereas culturally embedded practices may organize emotional expression, cultural continuity, social participation, and support simultaneously. Five gaps of special interest to Southwest China are identified. Gap 1: there is limited empirical research on traditional music and recovery-oriented psychological functioning among the Dai, Yi, Miao and Dong communities. Gap 2: previous work frequently examines music, cultural identity, social support, and resilience separately rather than within a single integrated framework. Gap 3: there has been limited integration of culturally embedded qualitative meanings with latent-variable associations, so statistical patterns and culturally situated meanings of music-related resilience have rarely been considered together. Gap 4: resilience has been insufficiently theorized as a culturally and relationally embedded process rather than an individual capacity. Gap 5: there is limited evidence regarding whether quantitative relationships among these constructs are experienced in the same way across cultural contexts, which is a question that a qualitative phase is well placed to address. To fill these gaps, the present study explores traditional music practice as a culturally embedded and multidimensional phenomenon among four ethnic minority groups, the Dai, Yi, Miao and Dong, in the Southwest region of China. The quantitative phase investigates the consistency of the observed covariance structure with a theoretically specified model involving the interrelationships between Traditional Music Engagement, Cultural Identity, Community Participation, Emotional Expression through Music, recovery-oriented psychological functioning, Perceived Social Support and Psychological Resilience. The qualitative phase provides a context for, and elaborates on, the quantitative results, and adds, if applicable, a level of complexity that is not built into the statistics, while directly addressing Gap 5 by exploring whether these associations were experienced in convergent or divergent ways across participants. The cross-sectional design of the quantitative research means that findings are interpreted as association, not as cause and effect.

### 2.6 Hypothesis development

While the present study incorporates select ideas from resilience theory and sociocultural theory, it does not define psychological resilience as a personal trait, or rigid personal quality. The focus of resilience theory is on the process by which individuals and groups respond to and adapt to adversity by mobilising psychological, social and environmental resources (Rogers et al., 2020). Sociocultural theory also suggests that human development and construction of meaning is intrinsically intertwined with participation in culturally organized practices, social relations and shared systems of knowledge (Nasir & Hand, 2006). Combined, these views point toward a Culturally Embedded Relational Resilience (CERR) framework, which proposes that resilience in culturally embedded communities may be expressed not solely as an individual psychological capacity but through the interaction of four dimensions: (1) emotional meaning-making, in which traditional music may provide culturally familiar ways to express, communicate, and regulate difficult emotions and to construct meaning around hardship; (2) cultural continuity, in which traditional music may connect individuals with ancestors, collective history, identity, cultural memory, and intergenerational transmission; (3) relational embeddedness, in which traditional music occurring within festivals, ceremonies, families, and community groups may generate perceived support through recognition, reciprocity, participation, and belonging; and (4) adaptive and future orientation, in which these resources may be associated with hope, persistence, coping, and adaptation. Within the Dai, Yi, Miao and Dong communities, traditional music is thus understood as a culturally situated practice through which these four dimensions may become interconnected.

Traditional music can at the individual-emotional level offer opportunities for emotional expression, emotional regulation, reflection and meaning making. Participation in music may provide other means to communicate a sense of grief, hope, fear, loss, or other experience that is hard to express in ordinary language. Repeated exposure to culturally familiar songs and performances can enable people to process challenging life events through familiar cultural stories and create coping supports related to psychological functioning in recovery (Sokira et al., 2022). Recovery-oriented psychological functioning (ROPF) refers in this study to self-perceived emotional restoration, coping, and perceived ability to continue functioning following difficult experiences; it is not a measure of clinical trauma recovery, PTSD remission, or treatment response, because the study did not include clinical trauma exposure or any type of trauma-related disorder diagnosis. The relationships proposed are, therefore, “associations” and not necessarily causal.

Traditional Music Engagement (TME) is defined as engagement in culturally meaningful music, such as folk songs, ceremonial music, festival music, instrumental playing, and music in the community. This engagement is not considered to be the same as clinical music therapy, as it does not necessarily involve professional music facilitation, structured therapeutic goal setting or a formal treatment procedure. The proposed framework conceptualizes traditional music as a culturally situated context in which emotional, cultural, and relational resources may be interconnected, and in which this potential psychological relevance may arise from musical experience, cultural value, joint involvement and collective memory. People with more active involvement in traditional music might have more opportunities to express and cope in a culturally meaningful manner. Thus, H1 proposed that Traditional Music Engagement (TME) is positively associated with recovery-oriented psychological functioning (ROPF).

Resilience at the cultural level can be related to cultural identity, collective memory, intergenerational learning and cultural continuity. Cultural Identity (CI) may play a role in giving a sense of belonging, continuity and meaning when there is a stressor or a change in a person’s culture. Those with strong feelings about cultural identity might situate challenging events within larger narratives about their ancestors, family history, shared traditions, and collective survival (Ungar, 2014). Cultural identification can also enhance self-awareness and a sense of support for culturally appropriate responses to challenges (Alam, 2025). These processes may be supported by traditional music, through the preservation of cultural knowledge and the linkage of past, present and future generations. Accordingly, H2 proposed that Cultural Identity (CI) is positively associated with recovery-oriented psychological functioning (ROPF).

On the relational and community level, engagement in co-created cultural activities can foster interpersonal relationships, sense of belonging, reciprocity and social resources. Community Participation (CP) refers to participation in events and culturally meaningful activities in which individuals interact, cooperate and maintain social relationships. Being part of a group can offer positive supports such as encouragement, help, emotional support, and shared coping (Talò et al., 2014). Community engagement can also build social ties and community support in challenging times (Abramson et al., 2015). Thus, H3 proposed that Community Participation (CP) is positively associated with recovery-oriented psychological functioning (ROPF).

Emotional Expression through Music (EEM) is the practice of using music to communicate, process and regulate emotions. Music can offer symbolic structures that are used to convey experiences which are hard to put into words (Juslin & Laukka, 2003). It may be used to express emotion in culturally appropriate ways and to convey feelings of sorrow, hope, remembrance and shared experience (Finnegan, 2012). These experiences may be linked to emotional regulation and to recovery-related functioning. Thus, H4 proposed that Emotional Expression through Music (EEM) is positively associated with recovery-oriented psychological functioning (ROPF).

Relational pathways can also be associated with traditional music. Engagement in music can include communication, cooperation, synchronisation, mutual recognition and shared emotional experience. These processes could reinforce participants’ perceptions of the availability of support from family members, friends and community at large (Vougioukalou et al., 2019). It can then be proposed that traditional music activity is positively associated with higher perceived social support. Therefore, H5 proposed that Traditional Music Engagement (TME) is positively associated with Perceived Social Support (PSS).

Cultural identity can also be related to social inclusion and relational belonging. A feeling of belonging to a common set of cultural beliefs and activities can help to promote recognition, acceptance and engagement in cultural groups (Walton et al., 2012). Cultural affiliation may enhance interpersonal relations and help to strengthen perceptions of others’ understanding and support (Schaafsma, 2011). Hence, H6 proposed that Cultural Identity (CI) is positively associated with Perceived Social Support (PSS).

Community Participation may provide direct opportunities to build relationships and reciprocal support networks. Collective cultural participation could heighten social interaction and enhance access to emotional, informational and practical resources (Letcher & Perlow, 2009). Community involvement may also foster shared responsibility and shared well-being (Galloway, 2006). Thus, H7 proposed that Community Participation (CP) is positively associated with Perceived Social Support (PSS).

Through listening and sharing music, empathy, trust and understanding may be fostered. Emotional communication using culturally familiar songs and performances can be received and recognized by others and evoke some of their own experiences, helping to validate feelings and foster a sense of social belonging (Saarikallio & Baltazar, 2018). Based on this reasoning, H8 proposed that Emotional Expression through Music (EEM) is positively associated with Perceived Social Support (PSS).

The outcome of the proposed multi-level framework is Psychological Resilience (PR), defined as adaptive psychological functioning in the face of adversity, not just an individual trait. Recovery-oriented psychological functioning may be associated with greater emotional balance and coping after challenging experiences, and with adaptation (Iacoviello & Charney, 2014). Therefore, H9 proposed that recovery-oriented psychological functioning (ROPF) is positively associated with Psychological Resilience (PR). Perceived Social Support can offer emotional and practical support and relational resources during hardship that may support adaptation (Schwarzer & Knoll, 2007; Sippel et al., 2015). Hence, H10 proposed that Perceived Social Support (PSS) is positively associated with Psychological Resilience (PR).

**Figure 1:**
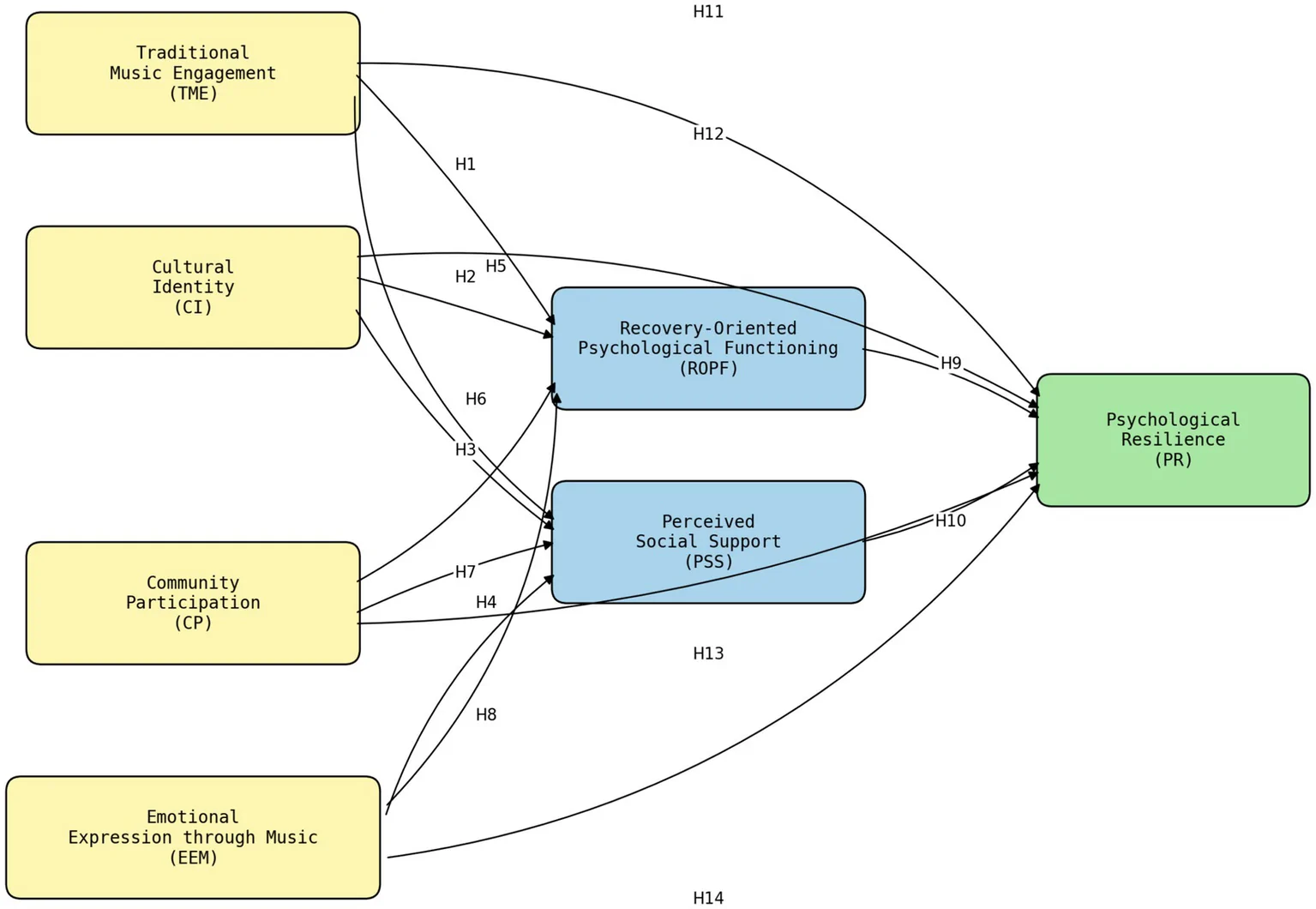
Conceptual framework diagram.

Traditional music-related variables could also be directly associated with resilience through their emotional, cultural and relational dimensions. Thus, H11–H14 proposed positive associations between Traditional Music Engagement (TME) and Psychological Resilience (PR), Cultural Identity (CI) and Psychological Resilience (PR), Community Participation (CP) and Psychological Resilience (PR), and Emotional Expression through Music (EEM) and Psychological Resilience (PR), respectively. Last, H15 examined whether recovery-oriented psychological functioning and perceived social support statistically accounted for indirect associations between the traditional music-related constructs and psychological resilience. Because all quantitative measures were collected cross-sectionally, these indirect associations are interpreted as covariance patterns consistent with the proposed model rather than evidence of temporal mediation, reflecting emotional and relational processes that may be culturally embedded in musical engagement. The proposed framework offers an integration of individual-emotional, cultural and relational aspects of resilience. It enriches individual-centred accounts by acknowledging that adaptive functioning can be situated within cultural continuity, community participation, shared meaning, and community relationships. The conceptual framework and hypothesized relationships among the constructs of this study are shown in Figure 1. Overall, the proposed framework integrates individual-emotional, cultural, and relational dimensions of resilience. It extends individual-centred accounts by recognising that adaptive functioning may be embedded in cultural continuity, collective participation, shared meaning, and community relationships.

The research aims and hypotheses have been systematically coordinated to correspond to the proposed multilevel approach. Aim 1 involving investigation of correlations of Traditional Music Engagement (TME), Cultural Identity (CI), Community Participation (CP), and Emotional Expression through Music (EEM) with recovery-oriented psychological functioning (ROPF) has been covered by H1-H4. Aim 2 related to investigation of relationships between TME, CI, CP, and EEM and Perceived Social Support (PSS) has been covered by H5-H8. Aim 3 including investigation of correlations of ROPF and PSS with Psychological Resilience (PR) has been addressed by H9 and H10. Aim 4 involved investigation of direct correlations between TME, CI, CP, EEM, and PR and has been covered by H11-H14. Aim 5 concerned investigation of indirect correlations between traditional music-related variables and PR via ROPF and PSS has been covered by H15.

## 3 Methodology

### 3.1 Research design

A sequential explanatory mixed methods design was used in this study to investigate the relationships between traditional music practices and psychological resilience among the Dai, Yi, Miao and Dong ethnic minorities in Southwest China. The research was done in two interrelated phases. First, the quantitative survey data was analysed to determine if the noted relationships between Traditional Music Engagement, Cultural Identity, Community Participation, Emotional Expression through Music, recovery-oriented psychological functioning, Perceived Social Support and Psychological Resilience were consistent with the proposed theoretical model. Second, the qualitative interviews were used to provide context, expand and clarify the quantitative patterns. Integration occurred at the stage of interpretation, through joint consideration of the statistical results and the participants’ culturally situated accounts. The mixed methods design was suitable because traditional music is embedded in an emotional, cultural, relational, ceremonial and community environment that cannot be captured solely quantitatively. The quantitative component was a cross-sectional survey. Thus, structural relationships were not taken to represent causal effects, but rather statistical associations. SEM allowed simultaneous estimation of relationships between latent constructs and controlled for measurement error, but did not imply either temporal precedence or causality. Likewise, statistically significant indirect relationships were considered patterns consistent with the proposed model, not evidence that traditional music influences recovery-oriented functioning, social support or psychological resilience. A hybrid deductive–inductive thematic analysis was conducted for the qualitative phase. Constructs from the quantitative framework were used as sensitising concepts, while inductive coding allowed participants’ accounts to introduce meanings not represented in the quantitative model. This was not conducted or reported as a grounded theory study.

### 3.2 Study setting and participants

This study was carried out in Xishuangbanna Dai Autonomous Prefecture (Yunnan Province), Liangshan Yi Autonomous Prefecture (Sichuan Province) and Qiandongnan Miao and Dong Autonomous Prefecture (Guizhou Province). These sites were deliberately chosen as traditional music continues to be used in a variety of cultural contexts for transmission, ceremonial use, festivals, community events, collective performances and intergenerational music. This study was conducted on ethnic minority groups of Dai, Yi, Miao and Dong people. The term ethnic minority communities has been used in accordance with the historical, social and academic context in China. While literature on Indigenous resilience was used to shape parts of the conceptual framework the participating groups were not directly equated with populations who have different political and colonial histories. The participants were chosen based on three criteria: being self-identified as Dai, Yi, Miao and Dong; residing in a certain community; and having engaged in traditional music activities for at least a year. The traditional ways of music engagement were in the form of folk songs, chants of ceremonies, instrumental music, performance of festivals, collective singing and music activities within community. The practices were not regarded as routine type clinical music therapy interventions but rather as culturally normal activities in individuals. No prior trauma exposure, clinical trauma diagnosis or trauma treatment was required. As such the construct previously considered to be “Trauma Recovery” was redefined as “Recovery-Oriented Psychological Functioning,” which included emotional recovery, coping skills, and how one perceived they would fare through difficult times moving forward. It was not used as a clinical evaluation of trauma recovery.

### 3.3 Sampling and data collection

The quantitative phase employed a multistage, stratified, community-assisted purposive sampling strategy. The three prefectures were selected purposively because they contained active and culturally significant traditional music practices. Following the sampling plan, six counties were selected, and communities within these counties were sampled proportionally to improve representation across locations and ethnic groups. Local community leaders and cultural organisations facilitated access, identified appropriate recruitment sites, and informed potentially eligible adults about the study. Participation was voluntary. Because recruitment was supported by community gatekeepers, the sampling process was not fully random. Eligibility required at least 1 year of traditional music engagement; the study therefore does not provide a comparison with adults who have little or no involvement in traditional music, and the findings describe associations within a population already connected to traditional musical practices rather than estimating differences between music-engaged and non-engaged community members. The target sample of 520 participants was determined using the general guideline of approximately 10 observations per estimated structural-model parameter, considering the complexity of the seven-construct latent-variable model and the requirements of structural equation modelling. After data screening, 17 questionnaires containing substantial missing information were excluded, resulting in 503 complete cases and a completion rate of 96.7%. Although the achieved sample supported estimation of the specified model, the original sample-size rationale was based on a rule-of-thumb approach rather than an *a priori* SEM power analysis, and this limitation should be considered when interpreting smaller structural coefficients and indirect associations; the rule-of-thumb approach is less objective than formal statistical power estimation. Future large-scale studies should use software such as G*Power or simulation-based SEM power analyses to determine sample requirements from anticipated effect sizes, statistical power, model complexity, and significance thresholds. Furthermore, community-assisted recruitment may have overrepresented culturally active, socially connected, visible, or research-willing individuals. Accordingly, the findings should not be assumed to represent all Dai, Yi, Miao, and Dong adults, particularly those with limited traditional music participation or weaker connections to cultural organisations.

The quantitative data were gathered from March to July 2025 using face-to-face surveys at community centres, cultural centres, village meeting places and other appropriate community venues. Questionnaires were administered by trained bilingual research assistants who were able to clarify questions if needed but did not influence the participant’s responses. The research ethics committee of the host university gave ethical approval. All participants were given information about the purpose of the study, confidentiality, voluntary participation, and rights to withdraw, which was followed by written informed consent. The analytical data did not contain any personally identifying information. The data collected has been uploaded in open database with URL https://github.com/boxcode59-source/Indigenous-folk-music-and-trauma-recovery.

### 3.4 Measurement development and cultural adaptation

The questionnaire used was structured in 7 constructs consisting of 4 independent variables, 2 mediating variables and 1 resulting variable. The independent variables were Traditional Music Engagement, Cultural Identity, Community Participation and Emotional Expression through Music. Recovery-oriented psychological functioning and Perceived Social Support were proposed mediators, and Psychological Resilience was the outcome. A final questionnaire had 38 items with a five-point Likert scale from 1, strongly disagree, to 5, strongly agree. The items were adapted from the Music Engagement Scale created by Rosenberg et al. (2021) to create Traditional Music Engagement. Cultural Identity drew on the dimensions of cultural identity from the Aboriginal Resilience and Recovery Questionnaire. Community Participation was drawn from indicators of community engagement within the field of resilience studies, and Emotional Expression through Music was based on measures from the music psychology literature that assessed emotional regulation and musical experience. Items for recovery-oriented psychological functioning drew on elements from the Aboriginal Resilience and Recovery Questionnaire and related concepts outlined in the Harvard Trauma Questionnaire; these items were adapted to assess recovery-oriented psychological functioning rather than clinical trauma recovery, and no item was interpreted as evidence of PTSD diagnosis, trauma exposure, or clinical treatment response. The Multidimensional Scale of Perceived Social Support (Zimet et al., 1988) was adapted for the creation of Perceived Social Support. Adapted items from the Connor–Davidson Resilience Scale were used to assess psychological resilience. More than translation alone, cultural adaptation was undertaken. All items were examined for conceptual relevance, cultural appropriateness, clarity and fidelity to local understandings of music, identity, support, emotional expression and resilience. Items with culturally inappropriate examples, unfamiliar terms, or assumptions at odds with local practices were altered or deleted. The adaptation record, presented in full as Supplementary file S2, documents for each item the original source, the original wording, the adapted wording, the cultural review, and whether the item was retained, modified, or deleted. The full questionnaire is provided as Supplementary file S1. Translation was conducted by bilingual persons with relevant linguistic and cultural background, and independent back-translation was carried out by translators who were not part of the original translation. Differences between the original and back-translated versions were checked to detect conceptual ambiguity or cultural inconsistency, and revision issues were resolved by discussion and consensus among experts. The content relevance, clarity, cultural appropriateness and conceptual coverage were evaluated by five experts in ethnic minority studies, traditional music, psychology, measurement, and quantitative research. In a pilot study with a sample of 50 participants, item comprehension, response patterns, preliminary reliability and cultural appropriateness were assessed. Cronbach’s alpha coefficients for the pilot ranged from 0.812 to 0.924, indicating good internal consistency.

### 3.5 Quantitative analysis

- Quantitative analyses were conducted using IBM SPSS Statistics 29 and IBM SPSS Amos. Structural equation modelling (SEM) was selected as the primary inferential approach because the proposed framework comprised seven latent constructs, multiple direct and indirect associations estimated simultaneously, two theoretically specified intervening variables, and measurement error that could be explicitly modelled. Multiple regression analyses, where undertaken, were treated only as supplementary sensitivity analyses and were not used as the primary basis for hypothesis testing. Given the cross-sectional nature of the data, all structural pathways were interpreted as statistical associations consistent with the proposed theoretical model rather than as evidence of causal effects or temporal relationships.

Before estimating the measurement and structural models, the dataset was systematically screened to evaluate completeness, missing values, data-entry accuracy, univariate and multivariate outliers, distributional characteristics, and potential violations of the assumptions relevant to SEM and indirect-association analysis. The extent and pattern of missing data were examined, and questionnaires with substantial incompleteness were excluded during the screening process. Univariate outliers were assessed using standardised scores and inspection of frequency distributions, whereas multivariate outliers were evaluated using Mahalanobis distance. Observations identified as potentially influential were examined individually to determine whether they reflected data-entry errors, implausible response patterns, or valid but extreme participant responses. No cases were removed solely because their values were statistically unusual; exclusions were based on predefined data-quality criteria and substantial questionnaire incompleteness.

The distributional properties of the observed variables were assessed using descriptive statistics, histograms, normal probability plots, skewness, and kurtosis values. Because maximum-likelihood estimation may be affected by substantial departures from multivariate normality, the assessment considered both univariate distributional characteristics and multivariate behaviour. The data showed no evidence of severe non-normality that would preclude estimation of the proposed SEM. Linearity was evaluated through inspection of relevant bivariate relationships and residual patterns, while multicollinearity was assessed using interconstruct correlations and diagnostic statistics. The results indicated that the predictor constructs were related but did not demonstrate levels of overlap considered sufficient to make the structural estimates unstable. The assumptions of independence and model specification were addressed through the study design and the theoretically defined ordering of the constructs. Nevertheless, because cross-sectional data cannot establish temporal precedence, the proposed ordering was treated as a theory-guided representation rather than as an empirically demonstrated causal sequence.

Descriptive statistics, Pearson correlation coefficients, and internal-consistency estimates were calculated before the latent-variable analyses. Cronbach’s alpha coefficients were reported for the full sample and separately for the Dai, Yi, Miao, and Dong groups to assess the consistency of the measures across the participating ethnic communities. The measurement model was subsequently evaluated using confirmatory factor analysis (CFA). Standardised factor loadings, composite reliability (CR), and average variance extracted (AVE) were used to assess convergent validity. Discriminant validity was examined using both the Fornell–Larcker criterion and the heterotrait–monotrait (HTMT) ratio. Model fit was evaluated using the chi-square statistic and chi-square-to-degrees-of-freedom ratio, the comparative fit index (CFI), Tucker–Lewis index (TLI), incremental fit index (IFI), root mean square error of approximation (RMSEA), and standardised root mean square residual (SRMR). The evaluation of model adequacy considered the overall pattern of fit indices rather than relying on a single statistic.

To strengthen the evaluation of the proposed framework, theoretically plausible alternative models were estimated and compared before drawing conclusions regarding the hypothesised structural model. The proposed model was compared with a more parsimonious direct-effects model in which Traditional Music Engagement, Cultural Identity, Community Participation, and Emotional Expression through Music were specified as direct correlates of Psychological Resilience without the two intervening constructs. An additional alternative model examined whether the relationships involving Recovery-Oriented Psychological Functioning and Perceived Social Support could be represented through a simplified structure with fewer theoretically differentiated pathways. Where appropriate, model comparisons were based on overall fit indices, chi-square differences for nested models, and information criteria such as the Akaike information criterion (AIC) and Bayesian information criterion (BIC). The preferred model was selected because it provided a theoretically coherent and empirically acceptable representation of the observed covariance structure while avoiding unnecessary model complexity. The comparison of alternative models was intended to evaluate whether the proposed framework offered a better account of the data than plausible competing specifications; it did not establish that the selected model was uniquely correct or causally verified.

The primary structural model was specified as follows:


$$TR_{i}=\alpha_{0}+\alpha_{1}\mathrm{TM}E_{i}+\alpha_{2}CI_{i}+\alpha_{3}CP_{i}+\alpha_{4}\mathrm{EE}M_{i}+\varepsilon_{\mathrm{TR},i}$$

$$\mathrm{PS}S_{i}=\gamma_{0}+\gamma_{1}\mathrm{TM}E_{i}+\gamma_{2}CI_{i}+\gamma_{3}CP_{i}+\gamma_{4}\mathrm{EE}M_{i}+\varepsilon_{\mathrm{PSS},i}$$

$$\begin{array}{l} PR_{i}=\delta_{0}+\delta_{1}\mathrm{TM}E_{i}+\delta_{2}CI_{i}+\delta_{3}CP_{i}\\ +\delta_{4}\mathrm{EE}M_{i}+\delta_{5}TR_{i}+\delta_{6}\mathrm{PS}S_{i}+\varepsilon_{\mathrm{PR},i} \end{array}$$

Where denotes Traditional Music Engagement, denotes Cultural Identity, denotes Community Participation, denotes Emotional Expression through Music, denotes Recovery-Oriented Psychological Functioning, denotes Perceived Social Support, and denotes Psychological Resilience. The residual terms represent unexplained variation in the respective endogenous constructs. The structural paths were estimated simultaneously to account for the interrelationships among the variables and to reduce the limitations associated with analysing each pathway in separate regression models. TMECICPEEMTRPSSPR

Indirect associations were evaluated using bias-corrected bootstrap confidence intervals based on 5,000 resamples. Bootstrapping was not adopted because the study lacked an adequate sample size. The retained analytical sample of 503 participants was considered sufficient for the complexity of the proposed latent-variable model. Instead, bootstrapping was used because indirect effects are products of estimated pathway coefficients and their sampling distributions are frequently asymmetric or non-normal, even when the individual component paths are approximately normally distributed. Consequently, conventional normal-theory tests, such as tests based only on the product coefficient and its standard error, may provide inaccurate confidence intervals for indirect associations. Bias-corrected bootstrapping generates an empirical approximation of the sampling distribution by repeatedly resampling the observed data and is therefore recommended for evaluating indirect pathways in mediation-oriented SEM.

For example, the indirect associations involving Traditional Music Engagement were represented as:


$$IE_{\mathrm{TME}\to \mathrm{TR}\to \mathrm{PR}}=\alpha_{1}\delta_{5}$$

$$IE_{\mathrm{TME}\to \mathrm{PSS}\to \mathrm{PR}}=\gamma_{1}\delta_{6}$$

Equivalent procedures were applied to Cultural Identity, Community Participation, and Emotional Expression through Music. An indirect association was considered statistically significant when its 95% bias-corrected bootstrap confidence interval did not include zero. The bootstrap analysis was interpreted alongside the direct-path estimates, model-fit results, and assessment of the measurement model. Because the data were collected at one time point, the indirect pathways were described as statistically significant indirect associations rather than causal mediation effects. The results therefore indicate whether the observed covariance structure is consistent with the proposed theoretical pathways, but they do not demonstrate that traditional music-related experiences temporally preceded changes in recovery-oriented functioning, perceived social support, or psychological resilience.

### 3.6 Common method bias and measurement invariance

All quantitative variables were self-reports, obtained through a single period of data collection and measured with a standard format of responding. Common method variance was therefore considered as a possible source of inflation. Procedural safeguards included assurances of anonymity, use of neutral language, clear instructions, and statements that no right or wrong answers existed; respondents were urged to give personal opinions rather than socially desirable answers. From a statistical point of view, the proposed seven-factor measurement model was contrasted against a one-factor model. The poorer fit of the single-factor model provides evidence against a model in which all item covariance is adequately represented by one general factor; however, this comparison does not establish that common method variance is absent. Because all constructs were measured using self-report data collected at one time point, residual method-related covariance cannot be excluded.

Multi-group confirmatory factor analysis was used to assess measurement comparability in the Dai, Yi, Miao, and Dong samples. Configural invariance tested the presence of similar general factor structures. Metric invariance tested the equivalence of factor loadings and permitted comparisons of structural relationships. Scalar invariance tested the comparability of latent mean comparisons, while strict invariance was tested wherever possible. Increases in comparative fit index and root mean square error of approximation were considered along with overall fit indices. Partial invariance was considered wherever theoretically sound if full invariance could not be demonstrated. Means and path coefficients for the groups were not compared unless the appropriate level of invariance was demonstrated. Measurement invariance provides evidence regarding statistical comparability of the measurement structure; it does not establish equivalence of cultural interpretation.

### 3.7 Qualitative phase and integration

Purposeful maximum-variety sampling based on ethnicity, age, gender, and varying level of involvement in traditional music was applied during the qualitative research stage to those survey participants who agreed to take part in further interviews. In total, 32 interviews were carried out, with 10 Dai, 8 Yi, 7 Miao, and 7 Dong participants taking part. Since the qualitative phase was nested within the quantitative one, the former was not considered a stand-alone qualitative research.

Semistructured interviews lasted for about 60–90 min and were held in the language that was chosen by the participant. The interview questions included issues related to traditional music, hard times, expression of emotions, cultural identity, community activities, social relations, perceived support, coping and resilience. Interviews were audio-taped with consent, transcribed verbatim, translated if necessary, and analyzed with the help of NVivo 14 software. The full version of interview guide is attached as Supplementary file S3.

Data analysis applied hybrid deductive-inductive thematic approaches based on Strauss and Corbin. Sensitizing concepts were deducted from the quantitative model, although the coding process was kept open to unforeseen experiences. The meaning units were revealed in open coding, relationships among categories in axial coding, and higher-order themes were built during integrative coding. Two analysts independently coded transcripts, and discrepancies between their interpretations were discussed and negotiated. The intercoder reliability (Cohen’s kappa) was 0.86. Credibility, dependability, and confirmability were ensured by member checking, peer review, discussion, and audit trail. The coding system should be presented in Supplementary file S4.

Integration happened at the stage of interpretation by joint consideration of statistical dependencies and qualitative themes. Qualitative data were interpreted in order to support, enrich, or contradict quantitative results rather than confirm them. Attention was paid to convergent, divergent, and culturally-specific findings. Since the quantitative study design was cross-sectional and the qualitative sample was embedded into the quantitative design, the integrated conclusions should be interpreted carefully as contextual explanations of associations observed.

## 4 Results

### 4.1 Quantitative findings

A total of 520 people were contacted and recruited for the study at the chosen sites. After data cleaning, 17 responses were removed from the analysis due to lack of data or incomplete responses. Thus, the sample used in the analysis was 503 individuals. The results reported below are not indicative of any causation but rather the relationships among self-reports.

#### 4.1.1 Demographic characteristics

Table 1 presents the demographic profile of the 503 participants. The gender distribution was relatively balanced, with 261 female respondents (51.9%) and 242 male respondents (48.1%). Participants aged 30–39 years constituted the largest age group (29.2%), followed by those aged 18–29 years (23.5%) and 40–49 years (22.3%). Participants aged 50–59 years accounted for 15.1% of the sample, while respondents aged 60 years or above represented 9.9%.

**Table 1:** Demographic profile of respondents (*N* = 503).

Variable	Category	Frequency	Percentage (%)
Gender	Male	242	48.1
	Female	261	51.9
Age	18–29 years	118	23.5
	30–39 years	147	29.2
	40–49 years	112	22.3
	50–59 years	76	15.1
	≥60 years	50	9.9
Ethnicity	Dai	136	27.0
	Yi	128	25.4
	Miao	123	24.5
	Dong	116	23.1
Education	Primary or below	98	19.5
	Secondary	176	35.0
	College Diploma	141	28.0
	Bachelor’s and above	88	17.5
Residence	Rural	364	72.4
	Semi-urban	139	27.6

The sample included participants from the Dai (27.0%), Yi (25.4%), Miao (24.5%), and Dong (23.1%) ethnic groups. Although the four groups were represented in broadly comparable proportions, the sample was not designed to be nationally representative of all members of these populations. Most respondents had completed secondary education (35.0%), followed by a college diploma (28.0%), primary education or below (19.5%), and a bachelor’s degree or higher (17.5%). Most participants resided in rural communities (72.4%), whereas 27.6% lived in semi-urban areas. This distribution was consistent with the predominantly rural character of many of the selected communities and the study’s focus on locations in which traditional musical practices remained embedded in community, ceremonial, and cultural life.

The means, standard deviations, inter-construct correlations, and square root of the average variance extracted (AVE) are presented in Table 2. On the whole, the participants expressed relatively high levels of their involvement in the following constructs: Traditional Music Engagement (TME), Cultural Identity (CI), Community Participation (CP), Emotional Expression through Music (EEM), Perceived Social Support (PSS), and Psychological Resilience (PR). As seen from the statistics, the highest mean value pertains to the construct TME (M = 4.12, SD = 0.71) and is very close to the mean value of the construct PR (M = 4.11, SD = 0.66).

**Table 2:** Descriptive statistics, discriminant validity and correlations.

Variable	Mean	SD	1	2	3	4	5	6	7
1. TME	4.12	0.71	0.825						
2. CI	4.05	0.68	0.687***	0.807					
3. CP	3.96	0.75	0.611***	0.642***	0.788				
4. EEM	4.01	0.73	0.628***	0.594***	0.659***	0.821			
5. ROPF	3.89	0.77	0.654***	0.633***	0.582***	0.689***	0.836		
6. PSS	4.08	0.69	0.617***	0.601***	0.687***	0.571***	0.644***	0.811	
7. PR	4.11	0.66	0.663***	0.652***	0.593***	0.676***	0.719***	0.691***	0.855

The means of the four predictors were 4.05 (SD = 0.68) for Cultural Identity, 4.08 (SD = 0.69) for Perceived Social Support, 4.01 (SD = 0.73) for Emotional Expression through Music, and 3.96 (SD = 0.75) for Community Participation. The lowest mean was recorded for Recovery-Oriented Psychological Functioning (ROPF) with the mean of 3.89 (SD = 0.77), although this was higher than the midpoint on the five-point scale. In accordance with the refined definition of this concept, the variable ROPF is considered as an indication of self-perceived recovery-related functioning, including emotional restoration, coping skills, and ability to move on after negative experiences, rather than recovery from trauma per se.

All study variables showed a significant positive relationship with each other at *p* < 0.001 (correlation analysis). ROPF was highly correlated with Psychological Resilience (*r* = 0.719), and Perceived Social Support was highly correlated with Psychological Resilience (*r* = 0.691); thus, both recovery-oriented psychological functioning and perceived social support were strongly associated with psychological resilience.

**Figure 2:**
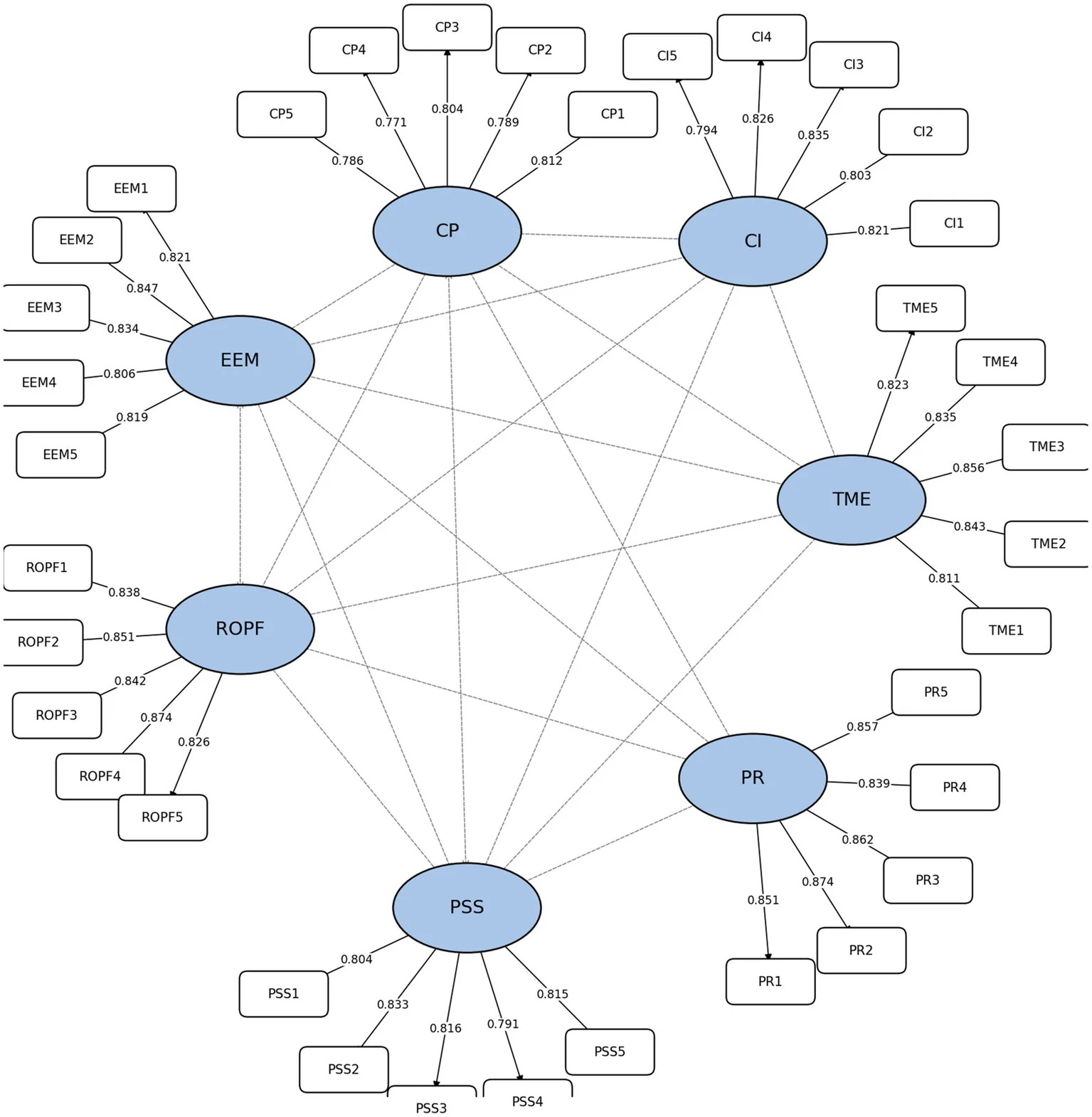
CFA measurement model.

The satisfactory psychometric properties were found for the measurement model (Table 3). The results of the reliability analysis indicated that Cronbach’s alpha values were in the range of 0.883 to 0.927, which is above the 0.70 guideline. The scores of the composite reliability ranged from 0.891 to 0.931, and the average variance extracted values ranged from 0.621 to 0.731, which indicated good convergent validity. The discriminant validity was also confirmed as the square root of the AVEs for each construct was higher than the inter-construct correlations. Moreover, all the factor loadings were statistically significant and higher than 0.70 (ranging from 0.771 to 0.874), suggesting high correlations between the observed indicators and the latent constructs (Figure 2).

**Table 3:** Reliability and convergent validity.

Construct	Cronbach’s *α*	CR	AVE
Traditional music engagement (TME)	0.908	0.914	0.681
Cultural identity (CI)	0.897	0.903	0.651
Community participation (CP)	0.883	0.891	0.621
Emotional expression through music (EEM)	0.906	0.912	0.674
Recovery-oriented psychological functioning (ROPF)	0.918	0.921	0.699
Perceived social support (PSS)	0.901	0.905	0.658
Psychological resilience (PR)	0.927	0.931	0.731

As there were four different ethnically diverse groups, reliability was assessed separately for each of the groups – Dai, Yi, Miao, and Dong. Group specific results are shown in Table 4. Internal consistency estimates were still satisfactory among all four groups. Thus, we can conclude that the reliability of the items was sufficient for the constructs within each ethnic community. However, reliability estimates alone cannot confirm that constructs have the same meaning within all groups.

**Table 4:** Internal consistency reliability by ethnic group.

Construct	Dai (*n* = 136) *α*	Yi (*n* = 128) *α*	Miao (*n* = 123) *α*	Dong (*n* = 116) *α*	Total sample (*N* = 503) *α*
Traditional music engagement (TME)	0.913	0.904	0.898	0.907	0.908
Cultural identity (CI)	0.902	0.891	0.894	0.899	0.897
Community participation (CP)	0.887	0.879	0.876	0.884	0.883
Emotional expression through music (EEM)	0.911	0.901	0.897	0.905	0.906
Recovery-oriented psychological functioning (ROPF)	0.923	0.916	0.911	0.918	0.918
Perceived social support (PSS)	0.907	0.896	0.893	0.902	0.901
Psychological resilience (PR)	0.932	0.925	0.921	0.928	0.927

The confirmatory factor analysis showed good model fit (Table 5), with a *χ*^2^/*df* of 2.31. The model achieved GFI = 0.924, AGFI = 0.907, CFI = 0.961, TLI = 0.956, IFI = 0.961, RMSEA = 0.051, and SRMR = 0.043, all of which satisfied established fit criteria. The same applies to the structural model, which showed good fit indices (Table 6) of *χ*^2^/*df* = 2.47; CFI = 0.955; TLI = 0.951; IFI = 0.955; RMSEA = 0.054; and SRMR = 0.047, indicating the proposed theoretical model as an appropriate framework for understanding psychological resilience within ethnic minorities.

**Table 5:** CFA model fit indices.

Fit index	Obtained value
*χ*^2^/*df*	2.31
GFI	0.924
AGFI	0.907
CFI	0.961
TLI	0.956
IFI	0.961
RMSEA	0.051
SRMR	0.043

**Table 6:** Structural model fit indices.

Fit index	Value
*χ*^2^/*df*	2.47
CFI	0.955
TLI	0.951
IFI	0.955
RMSEA	0.054
SRMR	0.047

In view of the fact that all quantitative variables were based on self-report measures obtained using identical surveys and the identical five-point Likert response scale, common-method variance was considered a potential problem that could lead to artificial inflation of the relationships observed. Several procedural steps were undertaken to minimize evaluation apprehension and response bias. Participants were reassured about the confidentiality of their responses, absence of right or wrong answers and were told to answer based on their experience. Sections of the questionnaire were grouped by construct and were worded in such a way as to be clear and non-evaluative.

Statistical analysis compared the proposed seven-factor measurement model against a single-factor model. The single-factor model showed considerably worse fit than the seven-factor model, indicating that a single common factor could not adequately explain the covariation among all observed measures. This finding provides evidence against a model in which all item covariance is represented by one general factor; however, it does not establish that common method variance is absent, since no statistical technique can fully remove the bias associated with a single-source, cross-sectional survey.

A multi-group confirmatory factor analysis approach was used to test the measurement comparability of the model across the four ethnic groups of Dai, Yi, Miao, and Dong. These tests were conducted using configural, metric, scalar, and strict invariance models in sequence. The findings from these tests are presented in Table 7.

**Table 7:** Multi-group measurement-invariance results.

Invariance Model	*χ* ^2^	*df*	CFI	TLI	RMSEA	SRMR	ΔCFI	ΔRMSEA
Configural invariance	2846.372	1,768	0.957	0.952	0.035	0.048	—	—
Metric invariance	2931.846	1,857	0.955	0.952	0.034	0.052	−0.002	−0.001
Scalar invariance	3042.118	1,946	0.952	0.950	0.033	0.055	−0.003	−0.001
Strict invariance	3196.754	2,061	0.948	0.947	0.032	0.058	−0.004	−0.001

The configural test showed acceptable fit in this model, meaning that the four ethnic groups had an equal general seven-factor structure. The metric invariance test was also seen to have acceptable comparative fit, suggesting that the relationships between the observed variables and their corresponding latent constructs were similar enough in all the groups.

The scalar invariance findings offered a basis for treating the item intercepts as reasonably comparable for interpreting latent mean differences. Nonetheless, because the current study focused on the pooled structural relationships rather than the comparison of latent means, the main importance of the invariance testing was to determine how uniformly the measurement model behaved across the four samples. Measurement invariance provides evidence regarding statistical comparability of the measurement structure; it does not establish equivalence of cultural interpretation. The findings indicate that the measurement model achieved reasonable statistical comparability, not that the underlying cultural meanings were identical.

However, the fit between the hypothesised structural model and the data was acceptable (Table 8). For the model obtained the following indicators were estimated: *χ*^2^/*df* = 2.47, CFI = 0.955, TLI = 0.951, IFI = 0.955, RMSEA = 0.054, and SRMR = 0.047. These indices suggested that the hypothesised structural model fits adequately the covariance structure. However, the goodness of fit does not imply that the relationships among variables are necessarily causal or ordered in time.

**Table 8:** Structural model fit indices.

Hypothesis	Path	*β*	SE	CR	*p*	Effect size (*f*^2^)	95% CI
H1	TME → ROPF	0.291	0.041	7.10	<0.001	0.125	[0.211, 0.372]
H2	CI → ROPF	0.244	0.045	5.42	<0.001	0.084	[0.158, 0.329]
H3	CP → ROPF	0.161	0.039	4.13	<0.001	0.041	[0.087, 0.238]
H4	EEM → ROPF	0.309	0.042	7.36	<0.001	0.138	[0.229, 0.392]
H5	TME → PSS	0.188	0.040	4.70	<0.001	0.052	[0.111, 0.267]
H6	CI → PSS	0.207	0.043	4.81	<0.001	0.061	[0.123, 0.287]
H7	CP → PSS	0.346	0.041	8.44	<0.001	0.173	[0.267, 0.423]
H8	EEM → PSS	0.132	0.039	3.38	0.001	0.028	[0.057, 0.208]
H9	ROPF → PR	0.421	0.048	8.77	<0.001	0.221	[0.336, 0.509]
H10	PSS → PR	0.288	0.046	6.26	<0.001	0.104	[0.201, 0.376]
H11	TME → PR	0.116	0.038	3.05	0.002	0.024	[0.042, 0.191]
H12	CI → PR	0.102	0.039	2.61	0.009	0.019	[0.026, 0.180]
H13	CP → PR	0.081	0.037	2.19	0.029	0.013	[0.008, 0.156]
H14	EEM → PR	0.138	0.041	3.37	0.001	0.031	[0.057, 0.219]

*R*^2^(ROPF) = 0.612; *R*^2^(PSS) = 0.547; *R*^2^(PR) = 0.734.

**Figure 3:**
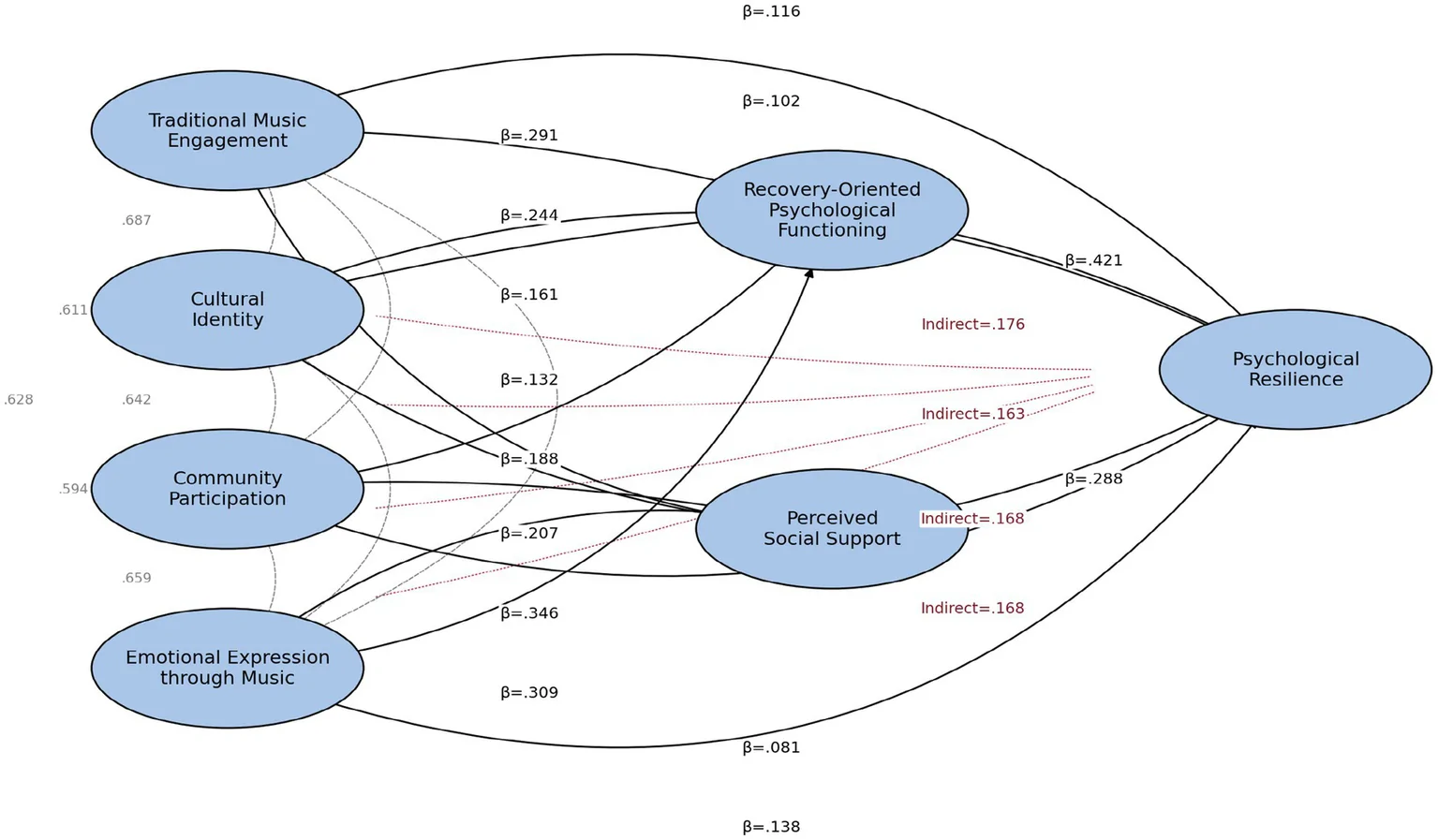
SEM pathway diagram.

The estimates for the structural paths are shown in Table 6 and depicted in Figure 3. All hypothesised relationships were found to be statistically significant. While this pattern corresponded to the hypothesised theoretical model, it is necessary to treat the positive results cautiously. First, all the constructs were assessed through the identical self-reported questionnaire, some constructs were highly correlated and no temporal order has been established due to the cross-sectional design. Therefore, the results could be influenced by the method of measurement and mutual effects of the constructs.

A significant positive association was found between TME and ROPF (*β* = 0.291, *p* < 0.001). Similarly, there was a significant positive association between ROPF and CI (*β* = 0.244, *p* < 0.001), CP (*β* = 0.161, *p* < 0.001), and EEM (*β* = 0.309, *p* < 0.001). Of the four predictors considered in the model, EEM had the highest standardized association with ROPF. This means that individuals who indicated more chances to express and regulate their emotions via traditional music were more likely to show recovery-oriented psychological functioning. All together, the four predictors accounted for 61.2% of the variance in ROPF (*R*^2^ = 0.612).

Also, all four music-related and sociocultural variables showed significant positive associations with PSS. The highest association coefficient was found for CP (*β* = 0.346, *p* < 0.001), then CI (*β* = 0.207, *p* < 0.001), TME (*β* = 0.188, *p* < 0.001), and EEM (*β* = 0.132, *p* = 0.001). These results imply that involvement in community-based cultural activities is significantly associated with the perception of emotional, relational, and communal supports. Collectively, the four predictors accounted for 54.7% of the variance in PSS (*R*^2^ = 0.547).

ROPF showed positive structural relationship with PR (*β* = 0.421, *p* < 0.001), having the highest direct structural relationship with resilience among all constructs. PSS had positive relationship with PR as well (*β* = 0.288, *p* < 0.001). Four predictor constructs maintained significant structural relationships with PR: TME (*β* = 0.116, *p* = 0.002), CI (*β* = 0.102, *p* = 0.009), CP (*β* = 0.081, *p* = 0.029), and EEM (*β* = 0.138, *p* = 0.001). Structural model accounted for 73.4% of the variance in PR (*R*^2^ = 0.734).

High level of variance explained should not be perceived only as indicator of prediction power. As the measures were taken at the same time using self-reported constructs, the levels of explained variance could have been affected by overlapping constructs and method effects. Replication with longitudinal, multi-source, or experimental methods would have been needed to establish whether such predictive strength can be observed under better-controlled conditions.

The analyses of the indirect associations were conducted via bias-corrected bootstrapping with 5,000 resamples. The results are summarized in Table 9. The statistical significance of the indirect associations was established when the corresponding 95% bootstrap confidence intervals did not include zero value.

**Table 9:** Mediation analysis (bootstrapping, 5,000 resamples).

Hypothesis	Indirect path	*β*	Boot SE	95% LLCI	95% ULCI
H15a	TME → ROPF → PR	0.122	0.021	0.084	0.167
H15b	CI → ROPF → PR	0.103	0.019	0.068	0.145
H15c	CP → ROPF → PR	0.068	0.016	0.039	0.102
H15d	EEM → ROPF → PR	0.130	0.022	0.092	0.177
H15e	TME → PSS → PR	0.054	0.013	0.031	0.084
H15f	CI → PSS → PR	0.060	0.014	0.036	0.090
H15g	CP → PSS → PR	0.100	0.018	0.068	0.138
H15h	EEM → PSS → PR	0.038	0.011	0.019	0.064

The indirect associations through ROPF proved to be statistically significant for TME (*β* = 0.122, 95% CI [0.084, 0.167]), CI (*β* = 0.103, 95% CI [0.068, 0.145]), CP (*β* = 0.068, 95% CI [0.039, 0.102]), and EEM (*β* = 0.130, 95% CI [0.092, 0.177]). The strongest indirect associations through ROPF were established for EEM and TME. It is possible to state that the associations of the four variables with PR were statistically consistent with the indirect effect mediated through recovery-oriented psychological functioning.

The indirect associations through PSS were also statistically significant. The estimated indirect association was the largest for CP (*β* = 0.100, 95% CI [0.068, 0.138]), followed by CI (*β* = 0.060, 95% CI [0.036, 0.090]), TME (*β* = 0.054, 95% CI [0.031, 0.084]), and EEM (*β* = 0.038, 95% CI [0.019, 0.064]). The relatively large indirect association between CP and PR through PSS suggested that the relationship in question could be explained by the participants’ perception of social support.

### 4.2 Qualitative findings: hybrid deductive–inductive thematic analysis

The qualitative sample was nested within the quantitative sample and consisted of 32 adult participants: 10 Dai, 8 Yi, 7 Miao and 7 Dong. Such sampling allowed for elaboration and contextualisation of participants’ answers to the survey. The qualitative phase was designed primarily for elaboration and contextualisation rather than independent triangulation. The two types of data were not fully independent, as all interview participants had completed the quantitative questionnaire prior to the interviews; participants’ responses to the questionnaire and their interviews may therefore have been more consistent because of their prior exposure to the survey constructs. The qualitative findings are accordingly used primarily to explain and elaborate on the quantitative results, rather than as an independent test of them. A hybrid deductive–inductive thematic analysis was conducted. Constructs from the quantitative framework, namely emotional expression, cultural identity, community participation, perceived support, recovery-oriented functioning, and resilience, were used as deductive sensitising concepts, while inductive coding allowed participants’ accounts to introduce meanings not represented in the quantitative model. Open coding was used to extract meaningful statements and initial concepts, axial coding was applied to explore relationships between categories, and integrative coding was used to organize inductively generated categories into the four higher-order themes. This analysis was not conducted, and is not reported, as an independent grounded theory study, since the interview guide was informed by the quantitative framework.

Four interrelated themes were developed: (1) Emotional Expression and Regulation through Traditional Music; (2) Cultural Continuity, Identity, and Meaning; (3) Collective Support and Community Cohesion; and (4) Adaptive Coping, Hope, and Future Orientation. The themes are presented as interpretive elaborations of the statistical associations rather than as direct evidence that traditional music caused psychological recovery.

#### 4.2.1 Theme 1: emotional expression and regulation through traditional music

Participants spoke of the traditional music as a culturally appropriate way to share, regulate, express, and eventually incorporate challenging emotions into individual and communal stories. Music was frequently referred to as a means of emotional communication that could not be accomplished in regular speech.

##### 4.2.1.1 Subtheme 1.1: expressing emotions that are difficult to verbalise

Traditional songs were often reported to be a means to share grief, anxiety, sadness and emotional distress without saying what they meant. “My grandmother’s song is more than a song, if I sing it, I will feel the sadness inside of me will be lighter, because the song has the emotion that words cannot convey,” said one Yi participant. (Participant 7, Yi community) A Dai participant similarly explained: “Sometimes I do not know how to speak about difficult things. When I sing the old songs, I feel that the song speaks for me, and I do not have to explain everything.” (Participant 12, Dai community) A Dong participant described music as a form of emotional recognition: “When people hear the same song, they understand the feeling behind it. You may not tell them what happened, but they know that the song carries sorrow and strength.” (Participant 24, Dong community) These accounts provide contextual detail for the positive association observed between EEM and ROPF. Participants described music as a culturally familiar form of emotional communication and, for some, emotional regulation.

##### 4.2.1.2 Subtheme 1.2: shared emotional release and regulation

Collective singing was also reported to build a context for dealing with difficult feelings with others. One Miao participant said: “When we sing together, it’s not just the one person who carries it, it’s the group that carries it, and after the singing I feel calmer.” (Participant 18, Miao community) A Yi participant (dai community) explained the emotional dynamic of a collective performance: “The music is not the eradication of memory, but rather of how powerfully the memory affects me after music. When I return to work after singing, I can do it with a clearer mind.” These narratives indicate that emotional regulation can take place through joint-activity, familiar music forms, and the mutual awareness of emotional experiences.

##### 4.2.1.3 Subtheme 1.3: remembering difficult experiences without becoming overwhelmed

Traditional music was not always comforting for some participants. Some participants mentioned that certain songs caused sadness, grief or distress as they were linked to death, loss of a loved one, difficult historical events or recollections of past struggles. One said: “Some of the songs of my ancestors remind me of people who are no longer there: I feel sadder, not less sad, when I hear the song, I have to give it time, before it can be comforting.” These differing accounts suggest that traditional music was not uniformly experienced as calming or supportive (Participant 16, Miao community), and that a song could soothe one person while causing another to cry (Participant 22, Dong community). Its psychological meaning appeared to depend on the type of song, the social context, the participant’s life history, and the moment of engagement. This divergence is significant: it indicates that music could function as either an emotion regulator or an emotion activator. The qualitative findings therefore qualified rather than simply confirmed the positive quantitative association between TME and ROPF: traditional music was not uniformly experienced as beneficial for all participants at all times, and its meaning varied according to musical form, social context, personal history, and current circumstances.

#### 4.2.2 Theme 2: cultural continuity, identity, and meaning

Traditional music was said to be a way to help participants to reconnect with their ancestors, their cultural identity, their collective history and a greater sense of meaning. Participants were often not only able to grasp personal challenges as individual experiences but also as collective stories.

##### 4.2.2.1 Subtheme 2.1: connection with ancestors and cultural continuity

Traditional music was often referred to as a link between the past and the present. “Traditional songs let us know who we are and when I am having problems in my life I sing traditional songs and I feel stronger because it reminds me of my ancestors,” said one of the Dai participants. These narratives provide contextual detail for the positive association between CI and ROPF (Participant 11, Dai community). “The songs remind me that I belong to something older than my own problems. My family and community carried these songs before me, and I am part of that continuing story.” (Participant 8, Yi community) “When the older people teach the songs, they are also teaching us how to remember. The music connects generations.” (Participant 23, Dong community) Participants’ accounts suggest that recovery-oriented psychological functioning may be related to cultural identity through a sense of continuity and belonging in a broader context, which may help participants situate difficult experiences.

##### 4.2.2.2 Subtheme 2.2: cultural pride and collective historical meaning

There was also mention by the participants that traditional music was a source of cultural pride and collective confidence. A Miao participant said: “As young people play our songs, it’s like we have also encountered problems in our past, and we are encouraged to overcome our problems because our culture is still alive.” As one of the Dai people explained (Participant 20): “The songs remind us that difficulties have been a regular occurrence in history, but so have been survival and continuity.” (Participant 3, Dai community) These accounts suggest that cultural meaning may function as a resource for coping, by situating challenging experiences within a broader narrative of continuity, endurance, and shared survival.

##### 4.2.2.3 Subtheme 2.3: intergenerational transmission

Resilience often was associated with a sense of responsibility to maintain and pass on traditional music. One participant said, “Older people teach me the songs and when I’m learning it, I feel that I have to carry it on for them.” This extended beyond affirmation of identity, as one participant (31, Dong) said, “When children learn the songs, they learn where they come from. This gives the community confidence about the future.” (Participant 14, Yi) Cultural transmission was also understood as an activity in the future, both as an activity that linked an individual with well-being to the continuity of the community.

#### 4.2.3 Theme 3: collective support and community cohesion

The importance of conventional music as a social practice that enabled participation, reciprocity, recognition and belonging was described. Support via sharing of musical activity was a common form of community support, rather than formal support-seeking.

##### 4.2.3.1 Subtheme 3.1: shared musical participation

Festivals, rehearsals, ceremonies and community performances were described as opportunities for social interaction for participants. A Dai participant said: “Other people listen and understand what you are saying when you sing together, you do not have to say it to them directly, you have common experiences, if it is difficult, the music can bring people together, the support can start before people ask for help.” (Participant 29, Dai community) These reports are in line with the quantitative result that CP was most strongly associated with PSS. Participation may be correlated with the sense of support due to the repeated opportunities for interaction and recognition.

##### 4.2.3.2 Subtheme 3.2: reciprocity and community recognition

Participants defined support as mutual and not unidirectional. One Dong participant explained: “If someone is having trouble, they do not give them advice directly, they invite them to participate in the activity, listen to them, and ensure that they are included.” One Miao person (Participant 27, Dong community) said: “It is important to be recognized by the group because when they ask you to sing or do something you feel you still have a place in the community.” Based on these reports, it seems that perceived support can be expressed in more than just providing emotional support, as it may include participation, recognition, and opportunities to contribute.

##### 4.2.3.3 Subtheme 3.3: reduced isolation and strengthened belonging

Collective music often was linked to decreased loneliness for participants. A Yi participant said: “When I face difficulties alone, they are hard, when I join the music group, I remember that there are others there also. The qualitative results thus indicate that community music may relate to resilience in part by relational resources, collective participation and decreased feelings of social isolation (Participant 15, Dai community).

#### 4.2.4 Theme 4: adaptive coping, hope, and future orientation

Participants said that traditional music helps in hope, perseverance, psychological flexibility and responsibility towards the coming generations. Resilience was defined as being a process of adaptation, not the absence of distress.

##### 4.2.4.1 Subtheme 4.1: hope and perseverance

Often times, traditional songs were seen as reminders of the sufferings that could be borne. One of the Dai participants said: “The songs are about people suffering and struggling; when I hear them, I think that it is not forever like that and that if they persevere, they will be able to overcome the difficulty.” These accounts provide contextual detail for the positive association between ROPF and PR (Participant 15, Dai community). “The music does not tell us to forget hardship. It teaches us to continue while carrying the memory.” Participants described recovery-oriented functioning as the ability to incorporate challenging events in a way that allowed them to hold both hope and everyday continuity.

##### 4.2.4.2 Subtheme 4.2: responsibility toward younger generations

Cultural transmission was a common way of expressing future orientation. Resilience might relate not only to individual coping, but also to involvement in an ongoing culture-building process, as indicated by a Dong participant: “A sense of responsibility to pass it on to the next generation, a sense of responsibility to the culture, whether that’s in terms of personal coping or involvement in a continuing cultural project.” (Participant 31, Dong community).

##### 4.2.4.3 Subtheme 4.3: adaptation through emotional, cultural, and social resources

In all the interviews, adaptive resilience was conveyed as a process that grows out of the interplay of emotional expression, cultural meaning and social connection. The qualitative findings elaborated this integrated picture: emotional processing, cultural identity, community support, and future-oriented meaning were described by participants as interwoven rather than independent processes. (Participant 30, Dai community)

### 4.3 Mixed-methods integration

Convergence, expansion and divergence were used to integrate the quantitative and qualitative findings during interpretation. The joint display is presented in Table 10. Rather than relying on quotations solely to illustrate statistically significant paths, the integration process examined how the qualitative findings explained, expanded, and/or qualified the quantitative results. The findings were most consistent for community participation and perceived social support: the quantitative model indicated that CP was the strongest predictor of PSS, while the qualitative findings showed that festivals, collective performances, reciprocal participation and community recognition were the ways in which support became available, so the qualitative findings explained this association in a culturally grounded way. The qualitative findings also elaborated the associations of TME, EEM, and CI with ROPF and PR: participants described emotional expression, ancestral connection, cultural continuity and collective historical meaning as processes that may help account for these positive associations. There were also divergent accounts, in which some songs evoked grief, anguish, or difficult memories; this divergence qualified the positive quantitative associations by showing that the same musical practices could carry both emotionally activating and emotionally calming meanings, depending on context. The integration cannot be interpreted as two independent sources converging on the same conclusion, because the qualitative sample is nested within the quantitative sample: the interviews primarily provided context for, and elaborated on, the survey findings. The mixed-methods meta-inference was that traditional music was experienced not as a uniform psychological intervention but as a culturally embedded practice whose potential relevance to adaptive functioning depended on interconnected emotional, cultural, relational, and future-oriented meanings. Quantitative associations identified the statistical relationships among these dimensions, whereas qualitative accounts illustrated how participants experienced those relationships in everyday community settings. Because the qualitative participants were drawn from the quantitative sample and completed the survey before interview, the qualitative findings cannot be regarded as independent replication of the quantitative results.

**Table 10:** Mixed-methods integration joint display.

Quantitative pattern	Qualitative evidence	Integration	Interpretation	Theoretical contribution
TME → ROPF	Emotional expression through shared singing	Expansion	Music was described as culturally familiar emotional communication	Emotional meaning-making
CI → ROPF	Ancestors, history, and continuity	Expansion	Cultural continuity contextualized the association	Cultural continuity
CP → PSS	Reciprocity, recognition, and inclusion	Convergence and expansion	Participation provided relational opportunities	Relational embeddedness
EEM → ROPF	Difficult emotions expressed through songs	Convergence	Emotional communication contextualized the association	Emotional meaning-making
ROPF → PR	Hope and persistence	Convergence	Adaptive functioning and resilience were closely related	Adaptive orientation
PSS → PR	Not being alone, sense of belonging	Convergence	Support represented a relational resource	Relational embeddedness
High TME but lower ROPF in some participants	Songs triggered grief	Divergence	Music was not uniformly experienced as beneficial	Context-dependence

The overall integration of the findings suggests that traditional music may be experienced as a culturally embedded resource operating across emotional, cultural, relational, and future-oriented dimensions. The findings provide preliminary support for the proposed model but should be interpreted with awareness of the cross-sectional nature of the data, the self-report measures, and the community-assisted sampling. Longitudinal and culturally comparative studies are needed to examine the stability of these associations over time and to explore the temporal or causal processes that may underlie them.

## 5 Discussion and implications

### 5.1 Overview of the integrated findings

The aim of this study was to explore the associations between TME, CI, Community Participation, EEM, recovery-oriented psychological functioning, perceived social support and psychological resilience among Dai, Yi, Miao and Dong communities in Southwest China. The integrated findings indicate that traditional music engagement was associated with a cluster of emotional, cultural, relational, and adaptive resources. The qualitative accounts suggest that these resources were experienced as interconnected rather than as isolated psychological processes. The four traditional-music constructs were positively associated with recovery-oriented functioning and perceived social support, and both were, in turn, positively associated with psychological resilience. The qualitative themes described music as a way to communicate difficult emotions, sustain cultural continuity, build community bonds, and hold onto hope.

### 5.2 Theoretical contribution: the culturally embedded relational resilience model

Individual-level resilience models are limited for this context because they tend to treat emotional coping, cultural belonging, and social participation as separate psychological processes, whereas the present findings suggest these processes were experienced by participants as interconnected. We therefore propose the Culturally Embedded Relational Resilience (CERR) model, comprising four interconnected dimensions. The first dimension, emotional meaning-making, is reflected in participants’ descriptions of traditional music as a culturally familiar means of expressing, communicating, and regulating difficult emotions and of constructing meaning around hardship; Traditional Music Engagement showed positive associations with recovery-oriented functioning and psychological resilience, and interviews indicated that music provided a culturally familiar means of expressing grief, sadness, fear and loss when verbal expression was difficult, consistent with research linking music-based activity to emotional regulation and adaptive coping (Feng & Wang, 2025). The second dimension, cultural continuity, is reflected in participants’ accounts of connecting with ancestors, collective history, identity, and intergenerational transmission through music. The third dimension, relational embeddedness, is reflected in the ways festivals, ceremonies, families, and community groups generated perceived support through recognition, reciprocity, participation, and belonging. The fourth dimension, adaptive and future orientation, is reflected in participants’ descriptions of hope, persistence, and responsibility toward younger generations. Together, these four dimensions constitute the proposed CERR model: resilience in culturally embedded communities may be expressed not solely as an individual psychological capacity but through the interaction of emotional meaning-making, cultural continuity, relational embeddedness, and future-oriented adaptive functioning, with traditional music representing one culturally situated practice through which these dimensions may become interconnected. The findings provide preliminary empirical support for the proposed model; they do not validate or prove it, since structural model fit demonstrates compatibility with the observed covariance structure rather than causality or temporal ordering. Research on bereaved families and conflict-affected communities also suggests that music interventions can be associated with emotional health and trauma-related outcomes (Alexandre et al., 2025; Wang et al., 2024); the current study extends this line of work to naturally occurring musical participation, rather than formal clinical music therapy, and these clinical findings should be read as contextual background rather than direct evidence for the effects of traditional music participation.

### 5.3 Cultural identity and collective meaning

Recovery-oriented psychological functioning, perceived social support and psychological resilience were positively associated with Cultural Identity. The qualitative findings showed that traditional music was described as connecting participants with ancestors, collective history, cultural pride and continuity across generations, and personal difficulties were sometimes framed within broader narratives of community resilience. Participants’ accounts suggest that music may provide a culturally familiar context for meaning-making by connecting personal difficulties with collective histories, identity, and continuity. This is consistent with evidence that cultural identity can be associated with resilience through meaning, belonging and social relationships (Zhou et al., 2025). These results resemble patterns reported in Western and Han Chinese studies, and this similarity should be considered with caution: shared patterns might reflect general-level processes such as emotional regulation, meaning-making and social support, but it is also possible that applying psychological constructs developed outside these communities overemphasized similarities and obscured culturally specific processes. Further research on folk song, ceremonial chant, festival music, instrumental tradition, and ritual performance is needed to examine whether distinct musical forms are associated with these processes in different ways.

### 5.4 Community participation and social support

Community Participation showed the strongest association with Perceived Social Support. Participants described festivals, group singing, ceremony, and shared observance as opportunities for recognition, reciprocity, emotional expression, and belonging. Participants frequently described musical participation in terms of belonging, recognition, reciprocity, and reduced feelings of isolation, and reinforced the sense that challenges would not be faced without the support of others. This finding aligns with research indicating that community musical activities can be associated with social cohesion, cooperation, and collective resilience during periods of uncertainty (Hanif & Sri Maruti, 2024; Netshivhambe, 2025). Perceived Social Support was positively associated with Psychological Resilience, suggesting a relationship between social support and adaptation; being understood, accompanied and connected with others was a recurring theme in participants’ accounts. However, the cross-sectional data cannot establish whether participation increases perceived support, whether perceived support increases participation, or whether more resilient or more socially connected individuals are more likely to participate. The qualitative findings also indicated that music was not uniformly experienced as beneficial: some participants reported sadness or distress in response to songs that evoked loss, difficult family histories, or painful memories. The meaning of traditional music therefore appeared to depend on musical form, personal history, and current circumstances, and could be supportive, emotionally activating, or both.

### 5.5 Methodological contribution and integration

The methodological contribution of the study lies in combining latent-variable modelling with qualitative contextualisation. Structural equation modelling examined several associations simultaneously while accounting for measurement error, while the qualitative analysis provided explanation, elaboration and complexity for these statistical patterns. For the music engagement–recovery-oriented functioning association, the qualitative findings pointed to emotional processing as a contextual explanation; for the community participation–perceived support association, they pointed to reciprocity; and for cultural identity, they pointed to ancestral connection. Because the qualitative sample was a subsample of the quantitative sample, the interviews are best understood as providing contextual elaboration rather than an independent test of the quantitative results. Constructs from the quantitative framework were used as deductive sensitising concepts, while inductive coding allowed participants’ accounts to introduce meanings not represented in the quantitative model; this hybrid deductive–inductive thematic approach was not conducted, and is not reported, as a complete grounded theory analysis.

### 5.6 Practical implications

Community-based traditional music activities can be viewed as a culturally meaningful complement to psychosocial support, cultural preservation and community well-being activities. Emotional expression, participation, belonging and cultural continuity may be fostered through festivals, community singing, intergenerational music education and locally led performances. These implications concern culturally responsive community programming rather than evidence for therapeutic efficacy. Culturally integrated music experiences could be considered as an addition to, not a substitute for, professional services, where co-created with community members, cultural practitioners, educators, community leaders, and mental health providers. The findings do not justify prescribing traditional music as a treatment for trauma, PTSD, or other clinical conditions.

### 5.7 Limitations and future research

This study has a number of limitations that should be considered when interpreting the findings. First, the cross-sectional design does not provide temporal precedence or evidence of cause and effect; direct and indirect pathways should be interpreted as associations rather than effects. Second, all quantitative constructs were self-reported within a single survey using similar response formats, raising the possibility of common method variance; while the seven-factor model fit the data better than a one-factor model, this comparison does not rule out common method variance. Third, social desirability bias may be particularly relevant here because traditional cultural practices may carry strong positive normative value within these communities. Fourth, community-assisted purposive recruitment may have overrepresented participants who were more socially connected or culturally active, so the findings should not be generalized to less-engaged community members. Fifth, eligibility required at least 1 year of traditional music engagement, so the study cannot estimate differences between music-engaged and non-engaged adults. Sixth, no clinical trauma exposure or diagnosis was assessed; the recovery-related construct reflects self-perceived recovery-oriented functioning, not clinical trauma recovery. Seventh, different musical forms, such as folk songs, ceremonial chants, and festival music, were combined within single constructs, which limits inferences about the specific musical forms underlying the observed associations. Eighth, the qualitative sample was nested within the quantitative sample and all interview participants completed the survey before being interviewed, so the qualitative findings provide contextual elaboration rather than independent triangulation of the quantitative results. Ninth, although the measurement model demonstrated statistical comparability across the four ethnic groups, measurement invariance does not establish that the underlying cultural meanings of the constructs were equivalent, and further cultural validation work is needed. Future research using longitudinal, comparative, participatory and intervention designs is needed to explore culturally specific pathways, examine distinct musical genres separately, and include community members with lower levels of traditional music engagement.

## 6 Conclusion

The current study explored the relationship between involvement in traditional music, recovery-oriented psychological functioning, perceived social support, and psychological resilience among the Dai, Yi, Miao, and Dong ethnic minority communities in Southwest China. Using structural equation modelling and a hybrid deductive–inductive thematic analysis, the results indicated that traditional music was associated with emotional, cultural, relational, and adaptive dimensions of psychological functioning. The mixed-methods findings suggest that its significance may arise from culturally embedded processes of emotional meaning-making, cultural continuity, community participation, and future-oriented adaptation, further contextualised by the qualitative findings on emotional expression, cultural continuity, community cohesion, and adaptive coping. These findings should be interpreted as associations rather than as evidence of therapeutic efficacy or causal effects: the quantitative measures were self-reported and collected at a single time point, which creates the potential for social desirability, recall bias, common method variance, and conceptual overlap, and the cross-sectional design does not permit causal or temporal inference. The recovery-related construct was defined as recovery-oriented psychological functioning rather than recovery from clinical trauma. Traditional music should therefore be regarded as a potentially important, culturally meaningful community resource, not as a proven treatment or intervention.

## Data Availability

The original contributions presented in the study are included in the article/Supplementary material, further inquiries can be directed to the corresponding author.
