# Supplementary material for: Traditional music, recovery-oriented psychological functioning, and psychological resilience among ethnic minority communities in Southwest China: a sequential explanatory mixed-methods study
Source: Front Psychol. 2026 Sep 3;17:1932535. doi: 10.3389/fpsyg.2026.1932535 (PMC13581667; doi:10.3389/fpsyg.2026.1932535)
Supplement: Supplementary file 1 [file Table_1.DOCX]

**Supplementary File S1**

**Response scale**

Please indicate how strongly you agree or disagree with each statement:

| **Score** | **Response** |
| --- | --- |
| 1 | Strongly disagree |
| 2 | Disagree |
| 3 | Neither agree nor disagree |
| 4 | Agree |
| 5 | Strongly agree |

**Section A. Demographic Information**

**A1. Gender**

☐ Male
☐ Female
☐ Prefer not to say

**A2. Age**

☐ 18–29 years
☐ 30–39 years
☐ 40–49 years
☐ 50–59 years
☐ 60 years or above

**A3. Ethnic community**

☐ Dai
☐ Yi
☐ Miao
☐ Dong
☐ Other: __________

**A4. Highest educational qualification**

☐ Primary education or below
☐ Secondary education
☐ College diploma
☐ Bachelor’s degree or above

**A5. Place of residence**

☐ Rural area
☐ Semi-urban area
☐ Urban area

**A6. How often do you participate in traditional music activities?**

☐ Never
☐ Rarely
☐ Sometimes
☐ Often
☐ Very often

**A7. Which traditional music activities do you participate in?**
*More than one answer may be selected.*

☐ Singing traditional songs
☐ Playing traditional musical instruments
☐ Participating in festivals or ceremonies
☐ Attending community music events
☐ Learning traditional songs from elders
☐ Teaching traditional music to younger people
☐ Listening to traditional music
☐ Other: __________

**Section B. Traditional Music Engagement (TME)**

**Concept:** The degree to which participants actively engage with, value, and experience personal meaning through traditional music.

**Adaptation basis:** Music engagement research, including Rosenberg et al. (2021), with cultural adaptation for traditional music practices. Rosenberg et al. examined associations between musical engagement and post-traumatic resilience.

| **Code** | **Statement** |
| --- | --- |
| TME1 | I regularly listen to, sing, play, or participate in the traditional music of my community. |
| TME2 | Traditional music is an important part of my everyday life. |
| TME3 | I actively seek opportunities to participate in traditional music activities. |
| TME4 | I feel personally connected to the traditional music of my ethnic community. |
| TME5 | Participating in traditional music gives meaning and value to my life. |

**Scoring:** Calculate the mean of TME1–TME5. Higher scores indicate greater traditional music engagement.

**Section C. Cultural Identity (CI)**

**Concept:** The extent to which individuals identify with, value, and maintain connection to their ethnic cultural heritage.

**Adaptation basis:** Cultural identity and cultural-strength dimensions derived conceptually from the Aboriginal Resilience and Recovery Questionnaire (ARRQ), with items reconstructed for the Dai, Yi, Miao, and Dong context. The ARRQ was developed as a culturally grounded, multidimensional strengths measure addressing personal, relational, community, and cultural resources associated with resilience, healing, and recovery.

| **Code** | **Statement** |
| --- | --- |
| CI1 | I have a strong sense of belonging to my Dai/Yi/Miao/Dong cultural community. |
| CI2 | The traditions and cultural values of my community are an important part of who I am. |
| CI3 | Traditional music helps me feel connected to my cultural heritage. |
| CI4 | I feel proud of the cultural traditions of my ethnic community. |
| CI5 | Maintaining our traditional music is important for preserving our cultural identity. |

**Scoring:** Calculate the mean of CI1–CI5. Higher scores indicate stronger cultural identity.

**Section D. Community Participation (CP)**

**Concept:** The extent to which individuals participate in collective cultural, musical, ceremonial, and community activities.

**Adaptation basis:** Indigenous community engagement and resilience literature, with local cultural adaptation.

| **Code** | **Statement** |
| --- | --- |
| CP1 | I participate in traditional music activities organized by my community. |
| CP2 | Traditional music activities help bring people in my community together. |
| CP3 | I feel that I have an active role in cultural or community events involving traditional music. |
| CP4 | I am willing to contribute my time or skills to preserve traditional music in my community. |
| CP5 | Traditional music activities strengthen cooperation among members of my community. |

**Scoring:** Calculate the mean of CP1–CP5. Higher scores indicate greater community participation.

**Section E. Emotional Expression through Music (EEM)**

**Concept:** The use of traditional music to recognize, express, regulate, communicate, and process emotions.

**Adaptation basis:** Music psychology research concerning emotional expression and emotional regulation through musical experiences.

| **Code** | **Statement** |
| --- | --- |
| EEM1 | Traditional music helps me express feelings that are difficult to express in words. |
| EEM2 | I use traditional music to cope with sadness, worry, or emotional stress. |
| EEM3 | Participating in traditional music helps me understand my emotions better. |
| EEM4 | Traditional music helps me release difficult or painful emotions. |
| EEM5 | I feel emotionally calmer or more balanced after participating in traditional music. |

**Scoring:** Calculate the mean of EEM1–EEM5. Higher scores indicate greater emotional expression and emotional regulation through music.

**Section F. Recovery-Oriented Psychological Functioning (ROPF)**

**Concept:** Self-perceived emotional restoration, coping, and perceived ability to continue functioning following difficult or distressing experiences. This construct is not a measure of clinical trauma recovery, PTSD remission, or treatment response.

**Important ethical note:** The questionnaire avoids requiring participants to describe specific traumatic events. The items assess perceived recovery-oriented functioning and coping rather than asking respondents to disclose trauma details.

**Adaptation basis:** Items were adapted from recovery-related dimensions of the ARRQ and from conceptual domains informed by the Harvard Trauma Questionnaire. Items were adapted to assess recovery-oriented psychological functioning rather than clinical trauma recovery; no item was interpreted as evidence of PTSD diagnosis, trauma exposure, or clinical treatment response. The ARRQ emphasizes strengths and resources associated with recovery, rather than treating resilience only as an individual psychological trait.

| **Code** | **Statement** |
| --- | --- |
| ROPF1 | I am able to regain emotional balance after difficult experiences. |
| ROPF2 | I feel that I have made progress in recovering from difficult experiences in my life. |
| ROPF3 | I am increasingly able to manage distressing thoughts or memories when they arise. |
| ROPF4 | I can continue with important daily activities even after experiencing emotional difficulties. |
| ROPF5 | I feel more hopeful about my future than I did during difficult periods of my life. |

**Scoring**: Calculate the mean of ROPF1–ROPF5. Higher scores indicate stronger recovery-oriented psychological functioning.

**Section G. Perceived Social Support (PSS)**

**Concept:** The extent to which participants perceive emotional, practical, and social support from family, friends, community members, and culturally meaningful relationships.

**Adaptation basis:** The Multidimensional Scale of Perceived Social Support (MSPSS) developed by Zimet et al. (1988). The original MSPSS assesses perceived support from family, friends, and significant others.

| **Code** | **Statement** |
| --- | --- |
| PSS1 | I have family members or relatives who are willing to help me when I need support. |
| PSS2 | I have friends or trusted people with whom I can share my concerns. |
| PSS3 | I can obtain emotional support from people in my community when I need it. |
| PSS4 | I feel supported and accepted by people who share my cultural traditions. |
| PSS5 | I know that there are people I can rely on during difficult times. |

**Scoring:** Calculate the mean of PSS1–PSS5. Higher scores indicate greater perceived social support.

**Section H. Psychological Resilience (PR)**

**Concept:** The ability to adapt, recover, persist, and maintain a sense of purpose when facing adversity or major challenges.

**Adaptation basis:** The Connor–Davidson Resilience Scale (CD-RISC). The original CD-RISC contains 25 items rated on a five-point response scale, and shorter versions also exist. The instrument is copyrighted; therefore, the following items are **culturally adapted indicators inspired by the resilience domains**, not a reproduction of the original CD-RISC items.

| **Code** | **Statement** |
| --- | --- |
| PR1 | I am able to adapt when important changes occur in my life. |
| PR2 | I can recover and continue moving forward after serious difficulties. |
| PR3 | I remain determined even when circumstances are challenging. |
| PR4 | I can find practical ways to deal with difficult problems. |
| PR5 | My cultural values and community connections give me strength during difficult times. |

**Scoring:** Calculate the mean of PR1–PR5. Higher scores indicate greater psychological resilience.

**Supplementary File S2**

**Item Adaptation, Translation, and Cultural Review Log**

**S2.1 Purpose of the Adaptation Process**

The adaptation process consisted of the following stages:

1. Review of the conceptual definitions and original items of the source instruments.
2. Identification of items that were culturally relevant, potentially inappropriate, unclear, or insufficiently contextualised.
3. Initial adaptation of item wording to the traditional music and community context.
4. Forward translation into Mandarin Chinese by qualified bilingual translators.
5. Independent back-translation into English.
6. Comparison of the original and back-translated versions.
7. Review by cultural and disciplinary experts.
8. Cognitive review and clarity assessment with members of the target population, where documented.
9. Pilot testing with 50 participants.
10. Final revision and preparation of the 38-item questionnaire.

The adaptation process aimed to achieve **conceptual equivalence rather than literal linguistic equivalence**. Changes were made only when they were necessary to improve cultural relevance, clarity, or contextual appropriateness while preserving the intended meaning of the underlying construct.

**S2.2 Construct-Level Adaptation Summary**

| **Construct** | **Source instrument/domain** | **Original items** | **Retained unchanged** | **Modified** | **Deleted** | **New culturally contextualised items** | **Final items** | **Main reason for adaptation** |
| --- | --- | --- | --- | --- | --- | --- | --- | --- |
| Traditional Music Engagement (TME) | Music Engagement Scale | 6 | 2 | 3 | 1 | 1 | 5 | To specify engagement with local folk songs, ceremonial music, festival music, and community musical practices |
| Cultural Identity (CI) | Aboriginal Resilience and Recovery Questionnaire-related cultural identity dimensions | 7 | 2 | 3 | 2 | 1 | 4 | To reflect ethnic heritage, ancestral traditions, local language, and cultural continuity |
| Community Participation (CP) | Indigenous community engagement indicators | 6 | 2 | 3 | 1 | 1 | 5 | To capture participation in festivals, collective music activities, cultural events, and intergenerational practices |
| Emotional Expression through Music (EEM) | Music emotion-regulation measures | 6 | 3 | 2 | 1 | 0 | 5 | To contextualise emotional expression within traditional rather than individualised music listening |
| Recovery-Oriented Psychological Functioning (ROPF) | ARRQ recovery domains and Harvard Trauma Questionnaire-informed domains | 8 | 2 | 4 | 2 | 1 | 5 | To assess recovery-oriented psychological functioning (emotional restoration, coping, and moving forward) rather than clinical trauma recovery; no item was retained as evidence of PTSD diagnosis, trauma exposure, or treatment response |
| Perceived Social Support (PSS) | Multidimensional Scale of Perceived Social Support | 12 | 5 | 4 | 3 | 1 | 6 | To include support from family, friends, community members, cultural groups, and trusted elders |
| Psychological Resilience (PR) | Connor–Davidson Resilience Scale | 25 | 4 | 4 | 17 | 1 | 6 | To retain adaptive coping while incorporating culturally and relationally grounded resilience |
| **Total** | — | **70** | **20** | **23** | **27** | **6** | **38** | — |

**S2.3 Translation and Back-Translation Procedure**

The questionnaire was initially prepared in English and translated into Mandarin Chinese by **[insert number] bilingual translator(s)** with expertise in **[insert relevant qualifications, such as psychology, cultural studies, public health, or translation]**. The translators were familiar with the linguistic and cultural contexts of the study areas.

An independent bilingual translator or translation team, who had not participated in the initial translation, back-translated the Mandarin version into English. The back-translated version was compared with the original English questionnaire to identify discrepancies involving:

- conceptual meaning;
- emotional intensity;
- cultural assumptions;
- ambiguity;
- idiomatic expressions;
- references to individual versus collective experience;
- terminology related to trauma, recovery, identity, support, and resilience;
- references to music participation that might not correspond to local practices.

Differences were reviewed by the research team and cultural experts. Revisions were made through consensus. The final wording was selected based on conceptual equivalence, cultural appropriateness, and participant comprehensibility rather than literal correspondence between languages.

Where local language assistance was required during data collection, trained bilingual research assistants explained the items using culturally appropriate terminology while preserving the meaning of the approved questionnaire. Any oral clarification was limited to explaining the intended meaning of the item and did not direct participants toward a particular response.

**S2.4 Expert Cultural Review**

Five experts reviewed the questionnaire. Their expertise included:

| **Expert** | **Area of expertise** | **Role in review** |
| --- | --- | --- |
| Expert 1 | Ethnic minority or cultural studies | Evaluated cultural relevance and contextual appropriateness |
| Expert 2 | Traditional music or ethnomusicology | Evaluated the representation of traditional music practices |
| Expert 3 | Psychology or mental health | Evaluated conceptual clarity and psychological relevance |
| Expert 4 | Quantitative research or psychometrics | Evaluated item structure and measurement relevance |
| Expert 5 | Community or culturally informed research | Evaluated community appropriateness and comprehensibility |

The experts evaluated each item using the following criteria:

1. Relevance to the intended construct;
2. Clarity of wording;
3. Cultural appropriateness;
4. Consistency with the experiences of Dai, Yi, Miao, and Dong communities;
5. Potential for misunderstanding;
6. Appropriateness of emotional and psychological terminology;
7. Suitability for a five-point Likert response format.

Items receiving substantive concerns were revised, removed, or flagged for pilot testing. The final decisions should be documented in the item-level adaptation log.

**S2.5 Pilot Study**

A pilot study was conducted with 50 participants from the target population. The pilot evaluated:

- clarity of questionnaire instructions;
- comprehensibility of item wording;
- appropriateness of the response options;
- cultural relevance;
- completion time;
- missing-response patterns;
- preliminary internal consistency.

Cronbach’s alpha coefficients ranged from **.812 to .924**, indicating satisfactory preliminary internal consistency. Pilot participants were invited to identify items that were unclear, difficult to answer, culturally unfamiliar, or inconsistent with their experiences. Feedback was reviewed before the final questionnaire was administered.

The final questionnaire contained 38 items. The complete final questionnaire is presented in **Supplementary File S1**.

**Supplementary File S3**

**Semi-Structured Interview Guide**

**S3.0 Methodological Transparency Note**

The 32 qualitative interviews reported in the main manuscript were conducted using the interview guide presented below in S3.1–S3.4. On review, several questions in this guide (particularly in Sections B, C, D, E, and F) ask participants directly about the quantitative constructs under investigation — for example, whether traditional music helps them express difficult feelings, cope with difficulty, feel connected to cultural identity, or contributes to resilience. This is a leading structure: it risks prompting participants to frame their accounts in terms of the researcher's expected constructs rather than allowing those constructs to emerge inductively from participants' own accounts.

We disclose this limitation transparently rather than retrospectively substituting a different, more open-ended guide as though it had been used to generate the existing dataset. Accordingly, the qualitative findings reported in the manuscript are interpreted as contextual elaboration of the quantitative associations rather than as independent qualitative confirmation of them, and the manuscript's Discussion and Limitations sections state this explicitly.

A revised, substantially more open-ended interview guide, designed to reduce leading questions and to allow participants to introduce emotion, coping, identity, community, support, and resilience in their own terms, is provided in S3.5 below for use in any future or supplementary data collection. It was not the instrument used to generate the interview data reported in the current manuscript.

**S3.1 Purpose**

The qualitative interviews were conducted after the quantitative phase to contextualise, elaborate on, and interpret the observed associations among traditional music engagement, cultural identity, community participation, emotional expression through music, recovery-oriented functioning, perceived social support, and psychological resilience.

The interview guide was informed by the quantitative model but was designed to allow participants to introduce experiences and meanings not represented in the survey. The interviews were not intended to test the quantitative hypotheses directly. Participants were encouraged to describe their experiences in their own words and to identify both positive and difficult aspects of traditional music participation.

**S3.2 Opening Script**

Thank you for agreeing to participate in this interview. We are interested in understanding your experiences of traditional music and its place in your personal, cultural, and community life. There are no right or wrong answers. We are interested in your own experiences and perspectives. You may choose not to answer any question, and you may stop the interview at any time. With your permission, the interview will be audio-recorded so that we can accurately understand your responses. Your identity will not be included in the research report.

**S3.3 Interview Questions**

**Section A: Personal and Cultural Background**

1. Please tell me a little about yourself and your connection to your ethnic community.

**Possible prompts:**

- How long have you lived in this community?
- What role does your ethnic culture play in your daily life?
- Which cultural traditions are most important to you?

2. What forms of traditional music are important in your community?

**Possible prompts:**

- Folk songs;
- ceremonial chants;
- instrumental music;
- festival music;
- collective singing;
- family or intergenerational music practices.

3. How did you first become involved in traditional music?

**Possible prompts:**

- Who introduced you to it?
- Did you learn from family members, elders, community members, or cultural teachers?
- Has your involvement changed over time?

**Section B: Traditional Music Engagement**

4. What does participating in traditional music mean to you personally?

**Possible prompts:**

- Is it mainly cultural, social, emotional, spiritual, recreational, or something else?
- What motivates you to participate?
- How often do you participate?

5. Can you describe a memorable experience involving traditional music?

**Possible prompts:**

- What happened?
- Who was present?
- How did you feel?
- Why was the experience meaningful?

6. Are there differences between listening to traditional music, performing it, singing with others, and participating in music during ceremonies or festivals?

**Possible prompts:**

- Do these forms affect you differently?
- Which forms are most meaningful to you?

**Section C: Emotional Expression and Emotional Healing**

7. How do you usually feel before, during, and after participating in traditional music?

**Possible prompts:**

- Happiness;
- calmness;
- comfort;
- sadness;
- grief;
- pride;
- connection;
- emotional release.

8. Does traditional music help you express feelings that are difficult to communicate through ordinary conversation?

**Possible prompts:**

- Can you give an example?
- Are there emotions that are easier to express through songs or music?
- Does music help you understand or organise your feelings?

9. Have you experienced difficult events, losses, major life changes, or periods of emotional distress that influenced your relationship with traditional music?

**Possible prompts:**

- You do not need to describe any experience you do not wish to discuss.
- Did music affect how you coped with the experience?
- Did music provide comfort, meaning, connection, or emotional expression?

10. Can traditional music sometimes bring back painful memories or difficult emotions?

**Possible prompts:**

- Are some songs associated with loss, grief, family separation, or difficult historical experiences?
- How do you respond when this occurs?
- Does the experience feel helpful, distressing, or both?

**Section D: Cultural Identity and Meaning**

11. How does traditional music influence your sense of belonging to your ethnic community?

**Possible prompts:**

- Does it make you feel connected to your ethnic identity?
- Does it remind you of your family, ancestors, homeland, or cultural history?

12. What cultural knowledge or values are transmitted through traditional music?

**Possible prompts:**

- Stories;
- history;
- moral values;
- language;
- customs;
- relationships;
- cultural responsibilities.

13. Do you think traditional music has changed in recent years?

**Possible prompts:**

- Modernisation;
- migration;
- younger generations;
- tourism;
- technology;
- changes in community participation.

14. How do you think the continuation of traditional music affects the future of your community?

**Section E: Community Participation and Social Support**

15. How does traditional music bring people together?

**Possible prompts:**

- Festivals;
- ceremonies;
- collective performances;
- family gatherings;
- community events.

16. Does participating in traditional music influence relationships among community members?

**Possible prompts:**

- Trust;
- cooperation;
- mutual assistance;
- belonging;
- respect;
- intergenerational relationships.

17. Can you describe a situation in which music participation helped you receive support from other people?

**Possible prompts:**

- Emotional support;
- practical assistance;
- advice;
- companionship;
- recognition.

18. Are there people who are excluded from, or less able to participate in, traditional music activities?

**Possible prompts:**

- Younger people;
- older people;
- migrants;
- women or men;
- people with limited cultural knowledge.

**Section F: Recovery-Oriented Functioning and Resilience**

19. In what ways, if any, has traditional music helped you cope with difficult experiences?

**Possible prompts:**

- Emotional recovery;
- maintaining hope;
- adapting to change;
- reconnecting with others;
- finding meaning.

20. Has traditional music influenced your confidence in dealing with future challenges?

**Possible prompts:**

- How?
- Can you provide an example?

21. What helps you continue moving forward when you experience difficulties?

**Possible prompts:**

- Family;
- community;
- cultural traditions;
- music;
- personal beliefs;
- economic or practical resources.

22. Do you think traditional music contributes to resilience? Why or why not?

**Section G: Integration and Reflection**

23. If you were explaining to someone outside your community how traditional music affects people’s well-being, what would you say?
24. Are there ways in which traditional music does not help, or may sometimes create pressure, sadness, conflict, or exclusion?
25. What do researchers often misunderstand about traditional music in your community?
26. Is there anything else you would like to share about traditional music, emotional well-being, cultural identity, community support, or coping with difficult experiences?

**S3.4 Closing Script**

Thank you for sharing your experiences. Some of the topics discussed may have involved difficult memories or emotions. How are you feeling after the interview? Is there anything you would like to add, clarify, or remove from the interview record?

**S3.5 Revised Semi-Structured Interview Guide (For Future or Supplementary Data Collection)**

Note: This guide was not used to generate the interview data reported in the main manuscript (see S3.0). It is provided as a methodological improvement for future or supplementary data collection, designed to reduce leading questions and allow participants to introduce concepts in their own terms.

**Title: Semi-Structured Interview Guide: Experiences and Meanings of Traditional Music in Everyday Community Life**

**Purpose of the Interview**

The interview is designed to explore participants' experiences and meanings associated with traditional music in everyday life. The interview is intentionally open-ended. Participants are not expected to describe traditional music as beneficial, harmful, therapeutic, or relevant to psychological well-being; positive, negative, mixed, or neutral experiences are equally relevant. The interviewer should avoid introducing psychological constructs such as resilience, trauma recovery, emotional regulation, social support, or cultural identity unless these topics arise naturally in the participant's account. Where a participant introduces one of these topics, the interviewer may use the corresponding optional probe.

**Opening and Rapport**

Could you tell me a little about yourself and your connection with your community? Could you tell me about your experience with traditional music? What kinds of traditional music are part of your everyday life? Can you describe a traditional music experience that you remember particularly well?

Optional probes: What was happening at the time? Who was involved? Where did it take place? What did you do? What made that experience memorable to you?

**Section 1. Meaning of Traditional Music**

What does traditional music mean to you? What place does traditional music have in your life? Has the meaning of traditional music changed for you over time? Are there particular songs, performances, instruments, ceremonies, or musical activities that are especially meaningful to you?

Optional probes: What makes that particular music meaningful? Can you tell me more about that? Is its meaning different in different situations? Does the meaning change depending on who is participating?

**Section 2. Experiences During Musical Participation**

Can you describe what you usually experience when you participate in traditional music? What kinds of thoughts, feelings, or memories, if any, come to mind during these experiences? Are there times when your experience of traditional music is different from what you expected? Can you describe a time when traditional music had a particularly strong effect on your experience or mood?

Optional probes: What happened? How did you experience that moment? What did you notice afterward? Was the experience positive, difficult, mixed, or neither? What do you think made the experience different?

**Section 3. Difficult Experiences and Coping**

When people in your community experience difficult periods in life, what kinds of things do they usually turn to? What, if anything, is the place of traditional music during difficult periods? Can you describe an occasion when traditional music was present during a difficult period in your life? What happened in that situation?

Optional probes: Did the music have any meaning for you at that time? Did your experience change during or after the musical activity? Did you talk with anyone about what you were experiencing? Was there anything about the music that made the experience more difficult? Was there anything that made it easier?

Interviewer instruction: Do not assume that traditional music helps participants cope. If participants say it was irrelevant, unhelpful, distressing, or both helpful and distressing, explore that account without redirecting it toward a positive interpretation.

**Section 4. Emotion and Expression**

How do people in your community usually express difficult feelings? What role, if any, does music have in expressing feelings? Can you describe a time when you expressed something through music that was difficult to express in another way? Are there situations in which music makes emotions stronger rather than easier to manage?

Optional probes: What kinds of emotions? What was the situation? Were other people involved? How did they respond? What happened afterward?

**Section 5. Culture, Identity, and Continuity**

What does participating in traditional music tell you about your community? What, if anything, does traditional music mean for your sense of who you are? Can you tell me about the relationship between traditional music and older generations in your community? What happens when traditional songs or musical practices are passed from older people to younger people? Are there traditions or meanings that you think are being preserved through music?

Optional probes: Family history? Ancestors? Language? Cultural knowledge? Community history? Responsibilities toward younger generations? Changes across generations?

Interviewer instruction: Do not ask whether music “strengthens cultural identity.” First ask what music means in relation to identity and culture.

**Section 6. Community and Relationships**

Where and with whom do you usually participate in traditional music? Can you describe what happens between people when they participate in music together? What kinds of relationships, if any, are connected with traditional music? Can you remember a time when participating in music brought people together? Are there situations in which participating in music creates distance, disagreement, exclusion, or discomfort?

Optional probes: Belonging? Recognition? Reciprocity? Cooperation? Conflict? Inclusion or exclusion? Help from others?

Interviewer instruction: Do not introduce “social support” unless the participant independently discusses receiving, giving, or seeking help.

**Section 7. Difficulties, Adaptation, and the Future**

When you think about difficult experiences, what does “moving forward” mean to you? What helps people continue with their lives after difficult experiences? What kinds of things give people hope about the future? Does traditional music have any place in these experiences? How, if at all, does passing traditional music to younger generations relate to your thoughts about the future?

Optional probes: Hope? Persistence? Confidence? Responsibility? Adaptation? Continuity? Future generations?

Interviewer instruction: Do not use the word “resilience” unless the participant uses it first or asks what the interviewer means by it. If the participant introduces resilience, ask “What does resilience mean to you?” rather than supplying a definition.

**Section 8. Contradictory or Difficult Experiences**

Are there times when traditional music does not feel helpful or meaningful to you? Can you describe a musical experience that was uncomfortable, painful, or difficult? Are there songs or musical practices that remind people of loss, conflict, difficult family experiences, or other painful events? How do people respond when music brings up difficult memories?

Optional probes: Does the experience change over time? Do people respond differently to the same song? Does the setting matter? Does who is present matter? Does the type of music matter?

**Section 9. Change Over Time**

Has your relationship with traditional music changed as you have grown older? Have changes in your community affected the way you participate in traditional music? How have migration, education, technology, urbanisation, employment, or generational change affected traditional music in your community? What do you think the role of traditional music will be in the future?

**Section 10. Participant-Led Closing**

Is there anything about traditional music and your experiences that we have not discussed but that you think is important? If you could explain to someone outside your community what traditional music means to people here, what would you want them to understand? Is there anything you would like to correct, clarify, or add to what you have told me?

**Interviewer Guidance — General Principles**

The interviewer should use open-ended questions before focused probes; encourage participants to provide concrete examples; allow participants to introduce their own meanings; avoid assuming that traditional music is beneficial; explore positive, negative, mixed, and neutral experiences; use participants' own terminology where possible; ask “Can you tell me more?” before introducing a conceptual interpretation; and distinguish the participant's interpretation from the researcher's interpretation.

**Avoiding Leading Formulations**

Instead of “Does traditional music help you cope?” ask “What, if anything, is the place of traditional music during difficult periods?” Instead of “Does music improve your emotional regulation?” ask “What do you experience emotionally when participating in traditional music?” Instead of “Does traditional music strengthen cultural identity?” ask “What does traditional music mean for your sense of who you are?” Instead of “Does traditional music provide social support?” ask “What kinds of relationships, if any, are connected with traditional music?” Instead of “Does traditional music contribute to resilience?” ask “When you think about difficult experiences, what does moving forward mean to you?” and only afterward explore whether traditional music has any place in that experience.

**Analytic Rationale**

This guide uses broad opening questions followed by optional probes. The quantitative constructs are treated as sensitising concepts rather than predetermined findings, which permits the qualitative phase to elaborate quantitative associations while allowing participants to introduce meanings, contradictions, and culturally specific processes that may not be represented in the quantitative model. The guide also explicitly invites accounts of negative, neutral, contradictory, and distressing experiences, because the quantitative findings represent statistical associations and cannot establish that traditional music is uniformly beneficial.

**Supplementary File S4**

**Qualitative Coding Framework**

The analysis involved:

1. Familiarisation with interview transcripts;
2. Initial open coding;
3. Development of preliminary categories;
4. Axial coding to examine relationships among categories;
5. Comparison of similarities and differences across participants and ethnic groups;
6. Integrative coding to develop higher-order themes;
7. Review and refinement of themes;
8. Comparison of qualitative themes with quantitative findings.

Two researchers independently coded the interview data. Coding differences were discussed and resolved through consensus. Cohen’s kappa was **.86**, indicating strong intercoder agreement. Member checking, peer review, and an audit trail were used to strengthen credibility, dependability, and confirmability.

**Deductive Sensitising Concepts and Inductively Generated Categories**

To address whether the qualitative themes were predetermined by the quantitative model, the coding framework distinguishes deductive sensitising concepts from inductively generated categories. The deductive sensitising concepts, drawn from the quantitative framework, were: emotional expression; cultural identity; community participation; social support; recovery-oriented functioning; and resilience. These concepts oriented the analysis but did not pre-specify participants' responses.

Inductively generated categories, developed from open and axial coding of the interview accounts rather than supplied in advance, included: emotional communication; collective emotional experience; painful memory activation; ancestral connection; cultural continuity; reciprocal participation; recognition; belonging; hope; and intergenerational responsibility. These inductive categories were subsequently organised, through integrative coding, into the four higher-order themes reported in the manuscript and summarised in the Coding Hierarchy below.

**Coding Hierarchy**

| **Final theme** | **Main categories** | **subcategories or codes** |
| --- | --- | --- |
| **Theme 1: Emotional Expression and Regulation through Traditional Music** | Emotional expression; emotional release; emotional regulation; comfort and soothing; processing difficult experiences | Expressing feelings through song; releasing sadness; calming emotional distress; feeling comforted; emotional catharsis; shared emotional experience; music as a safe expressive space; remembering difficult experiences |
| **Theme 2: Cultural Continuity, Identity, and Meaning** | Cultural belonging; cultural continuity; intergenerational transmission; collective memory; cultural pride; meaning-making | Feeling connected to ethnic identity; remembering ancestors; preserving language and heritage; learning from elders; cultural pride; continuity across generations; traditional music as cultural knowledge |
| **Theme 3: Collective Support and Community Cohesion** | Social connection; collective participation; mutual support; intergenerational relationships; belonging; community solidarity | Singing together; festival participation; feeling included; receiving support; strengthening trust; shared responsibility; community recognition; collective identity |
| **Theme 4: Adaptive Coping, Hope, and Future Orientation** | Coping capacity; hope; confidence; adaptability; future orientation; cultural continuity | Moving forward after difficulties; maintaining hope; confidence in facing challenges; adapting to social change; drawing strength from culture; supporting younger generations; sustaining traditions |
